# Comparative Analysis on Abnormal Methylome of Differentially Expressed Genes and Disease Pathways in the Immune Cells of RA and SLE

**DOI:** 10.3389/fimmu.2021.668007

**Published:** 2021-05-17

**Authors:** Qinghua Fang, Tingyue Li, Peiya Chen, Yuzhe Wu, Tingting Wang, Lixia Mo, Jiaxin Ou, Kutty Selva Nandakumar

**Affiliations:** ^1^ School of Pharmaceutical Sciences, Southern Medical University, Guangzhou, China; ^2^ Laboratory of Experimental Oncology, Department of Pathology, Erasmus Medical Center, Rotterdam, Netherlands; ^3^ Department of Science and Education, First Affiliated Hospital of Jinan University, Guangzhou, China

**Keywords:** rheumatoid arthritis, systemic lupus erythematosus, immune cells, methylation, bioinformatics analysis

## Abstract

We identified abnormally methylated, differentially expressed genes (DEGs) and pathogenic mechanisms in different immune cells of RA and SLE by comprehensive bioinformatics analysis. Six microarray data sets of each immune cell (CD19^+^ B cells, CD4^+^ T cells and CD14^+^ monocytes) were integrated to screen DEGs and differentially methylated genes by using R package “limma.” Gene ontology annotations and KEGG analysis of aberrant methylome of DEGs were done using DAVID online database. Protein-protein interaction (PPI) network was generated to detect the hub genes and their methylation levels were compared using DiseaseMeth 2.0 database. Aberrantly methylated DEGs in CD19^+^ B cells (173 and 180), CD4^+^ T cells (184 and 417) and CD14^+^ monocytes (193 and 392) of RA and SLE patients were identified. We detected 30 hub genes in different immune cells of RA and SLE and confirmed their expression using FACS sorted immune cells by qPCR. Among them, 12 genes (BPTF, PHC2, JUN, KRAS, PTEN, FGFR2, ALB, SERB-1, SKP2, TUBA1A, IMP3, and SMAD4) of RA and 12 genes (OAS1, RSAD2, OASL, IFIT3, OAS2, IFIH1, CENPE, TOP2A, PBK, KIF11, IFIT1, and ISG15) of SLE are proposed as potential biomarker genes based on receiver operating curve analysis. Our study suggests that MAPK signaling pathway could potentially differentiate the mechanisms affecting T- and B- cells in RA, whereas PI3K pathway may be used for exploring common disease pathways between RA and SLE. Compared to individual data analyses, more dependable and precise filtering of results can be achieved by integrating several relevant data sets.

## Introduction

As immune mediated diseases, the pathogenesis of rheumatic diseases, rheumatoid arthritis (RA) and systemic lupus erythematosus (SLE) is closely related to different immune cells (1). These cells secrete many pro-inflammatory factors and proteases that could destroy cartilage and bone (2). Chronic inflammation in the articular joints leads to joint and bone destruction in RA, whereas uncontrolled production of autoantibodies against nuclear antigens leads to systemic inflammation in SLE. Both of these diseases can easily be distinguished based on their phenotypes. Although standard diagnostic methods are available for RA and SLE, identifying sub-populations of patients for personalized medicine and sub-phenotypes of the disease, needs many different diagnostic methods that can also distinguish different phases of the disease activity. In this context it is of interest to mention that RA and SLE are considered as syndromes having a combination of different disease phenotypes. In addition, earlier studies (3–5) have shown that peripheral blood gene expression profiles and expression of a unique set of genes can differentiate responders from non-responders during biological therapy and also can help to predict responsiveness of therapy to support the clinical decision-making process. Although familial aggregation (6), genetic overlap (7–9), shared locus (10) and common molecular mechanisms (11, 12) between them have been reported, differences, especially in the non-MHC gene regions were also documented (13, 14) suggesting presence of shared and distinct loci and biological pathways among them (15), including pleiotropic genes (13).

B cells have both antibody-dependent and -independent roles in the pathogenesis of RA and SLE. Autoantibodies are not only important for clinical diagnosis but also act as inflammatory mediators together with complement and Fc*γ*R-expressing immune cells as downstream mediators of inflammation at the effector phase of disease development (16). Antibody-independent mechanisms include antigen presentation, regulation of T- cell and DC functions and, production of cytokines, which cause development of diseases (17). B cell targeted therapies were reported to be successful in the treatment of RA and SLE (18), while CD4^+^ T cells also contribute to initiation and perpetuation of diseases. A balance between T cell subgroups (Th1, Th2, Th17 and Treg) is important for maintaining normal immune function (19) and dysregulation of this balance was observed both in RA (20) and SLE (21, 22). Apart from B cells, targeting T cells has also proved to be beneficial in the treatment of these diseases (23). Similarly, monocyte contributes to the immune and inflammatory responses by their antigen presenting capacity, which regulates T cell differentiation and phagocytosis of pathogenic microorganisms. After entering the synovium, monocytes get differentiated into macrophages and produce pro-inflammatory factors, and they can also further differentiate into osteoclasts, and participate in bone destruction and resorption (24–26). Depletion of macrophages proved to be beneficial in treating RA (27). Also, defects in phagocytic function may be the basis for the pathogenesis of SLE. Lupus is initiated in mice, when they lack molecules related to clearing apoptotic cells (28). In addition, monocytes play an active role in accelerating inflammation and damage to glomeruli (29).

DNA methylation status is one of the critical parameters in predicting RA and other immune mediated inflammatory diseases. At present, many studies have proposed candidate genes that undergo methylation changes from B-, T- and synovial cells as well as monocytes as clinical markers in RA patients (30). However, studying the changes in the methylation profile alone will only reflect the methylation status at cellular level but will not take into account other changes present in closely related environmental and genetics factors. Moreover, methylation can also be easily altered by other factors, thus posing strong reliability issues. Hence, if changes in methylation profiles can be combined with other omics data, it will have greater significance as clinical markers. Therefore, we envisaged a combined analysis of methylation profile and transcriptomics data might have a more clinical relevance. Such aberrantly methylated genes in RA and SLE may be used as biomarkers or predictors of disease progression, and severity (31). Moreover, by analyzing the gene expression in RA and SLE, we can get new insights into sex-bias in the inflammatory functions, gene biotypes and co-expression patterns (32). However, until now, a combined analysis of gene expression and methylation profiling microarray in immune cells of RA and SLE has not been performed. Therefore, we collected relevant microarray data sets for B- and T-cells, as well as monocytes from these two diseases, and used a variety of bioinformatics tools for integrated analysis to detect exceptionally methylated differentially expressed genes (DEGs) and pathogenic pathways. Protein–protein interaction (PPI) networks were developed and hub genes were determined. Validity of these hub genes were confirmed by analyzing their expression by qPCR using FACS sorted immune cells from patients and healthy volunteers. In addition, we proposed certain genes as potential biomarkers for diagnosis based on receiver operating characteristic curve (ROC) analysis that need to be verified using large cohort of human samples. Moreover, distinct signaling pathways operating at cellular level in both RA and SLE were identified and discussed.

## Materials and Methods

### Data Collection

We checked the GEO database (https://www.ncbi.nlm.nih.gov/geo/) with several keywords to identify relevant data sets: (“Rheumatoid arthritis” OR “RA” OR “Arthritis” OR “Rheumatoid”) AND (“Systemic lupus erythematosus” OR “SLE” OR “lupus” OR “Erythematosus”) AND (Immune cells) AND (“B cell” OR “CD19+” OR “B lymphocyte”) AND (“T cell” or “CD4+” OR “T lymphocyte”) AND (“Monocyte” OR “CD14+” OR “Mononuclear cell” OR “Mononuclear leucocyte”). The screening criteria were as follows: 1) all cell sources consistent for pre-treatment status were selected; 2) arrays must have at least five patients per group; 3) more than 5,000 genes should be included in the GEO database; 4) the entry and organism were limited to “series,” and “Homo sapiens,” respectively.

### Detection of DEGs and DMGs

A series of matrix files from GEO data sets were used. After background noise was corrected, the raw data were normalized by Robust Microarray Analysis (RMA) procedure. Standardized procedures like filtering and log2-transformation, were performed. For data normalization and, to identify differentially expressed genes (DEGs) and differentially methylation genes (DMGs), the R package-limma (33) was used. The one-tailed p-value of each gene was used to identify its ranking in the list of genes, and the genes having p < 0.05 were taken as relevant DEGs or DMGs. We have used this p-value for our combined data analysis to avoid type I and type II errors.

### Function Enrichment Analysis

Gene Ontology (GO) annotations and Kyoto Encyclopedia of Genes and Genomes (KEGG) analysis of aberrantly methylated DEGs were done by using the database for annotation, visualization, and integrated discovery (DAVID) or Pathview (34) software. DAVID is an online free software, which provides functional annotation tools to analyze and understand the biological meaning behind the list of genes. Using this tool, we identified enriched GO terms to describe the role of genes and proteins in cells, and to fully describe their attributes in organisms and visualize the genes on KEGG pathway maps to study the metabolic pathways and gene functions within the cells. The results in.txt files were applied for visual analysis using GO Plot18 and ggplot2 packages in the R program.

### Analysis of Protein-Protein Interaction (PPI) Network

PPI networks for aberrantly methylated DEGs were created using “Search Tool for the Retrieval of Interacting Genes/Proteins” (STRING) database (http://stringdb.org/) for hypomethylated highly expressed genes and hypermethylated low expressed genes. The cutoff interaction score was set at 0.4 for visualizing PPI. Next, the “Molecular Complex Detection” (MCODE) in Cytoscape software 3.7.2 was performed to filter modules in the PPI network having MCODE score and the number of nodes more than 3. Cytoscape is an open-source software for not only visualizing molecular interaction networks and biological pathways but also it aids to integrate them with annotations and gene expression profiles. This software contains the plug-in MCODE, which we used to calculate the density of a node and its surrounding nodes by selecting a largest score value, followed by enrichment of the neighboring nodes to finally form a functional module. The gene functional enrichment analysis in each module was determined by using STRING having a p-value < 0.05. Top 10 hub genes were selected using Maximal Clique Centrality (MCC) in Cytoscape software, which is based on centrality, eccentricity and radiality. Moreover, Metascape was used to predict the biological characteristics and analyze the biological functions contained in the modules within PPI network (35).

### Methylation Gene Analysis

The human disease methylation database (DiseaseMeth 2.0, http://bioinfo.hrbmu.edu.cn/disease meth/) contains methylation modifications data derived from microarray and sequencing analysis as well as annotated data for DNA methylation status for several diseases (36). This resource includes the methylation data from Gene Expression Omnibus (GEO) and has experimental methylation-associated gene-disease associations, inferred methylation-associated gene-disease associations and potential methylation-associated gene-disease associations. We compared methylation levels of both RA, SLE and healthy individual’s hub genes using this web site.

### ROC Analysis

To identify the candidate genes for RA and SLE diagnosis, we used RStudio with pROC package to do ROC analysis (37). The area under the curve (AUC) was used to evaluate the sensitivity and specificity of each gene. The genes with AUC more than 0.7 and two-tailed p-value less than 0.05 were used to predict relevant biomarkers.

### Clinical Samples

The selection of RA patients is based on The 1987 ACR guidelines (38) and SLE patients is based on The 2019 SLE EULAR/ACR classification standards (39). In this study, we included 10 RA and 10 SLE patients as well as 10 healthy controls from first affiliated hospital of Jinan University. We have also collected patient’s age, gender, DAS28 score (for RA), SLEDAI score (for SLE) and other relevant clinical information including clinical lab findings such as erythrocyte sedimentation rate (ESR), levels of c-reactive peptide (CRP), anti-cyclic citrullinated peptide (CCP) antibodies, circulating immune complex (CIC), anti-ds-DNA and anti-Smith antibodies, rheumatoid factor, etc (see **Supplementary Table, ST1**). All the samples collected in this study were approved by the ethics committee of first affiliated hospital of Jinan University, Guangzhou, China (Ethical number (2017:17). All the blood samples were collected after written informed consent from individuals based on declaration of Helsinki. Procedures for using patient samples were approved prior to start of this study by the ethics committee of the first affiliated hospital of Jinan University. The screening sample criteria were as follows: 1) All the sample sources were from one hospital; 2) Each group contained a minimum of 10 patients; 3) Patients were newly diagnosed with RA or SLE. They were admitted as in-patients with a clear diagnosis having obvious clinical symptoms, and at their initial stage of disease with no other reported diseases; 4) Patient’s sample was collected immediately after the diagnosis became clear. All the patients were not treated with any medications before collecting the samples for this study.

### Flow Cytometry

The blood samples were centrifuged to collect the plasma. White blood cells were obtained after red blood cells were lysed and centrifuged at 1000 rpm. After incubating with Fc receptor blocking solution (Human TruStain FcX™, Biolegend, California, USA) for 30 min., the cells were further incubated with FITC anti-human CD19, APC anti-human CD4 and PE anti-human CD14 (Biolegend, California, USA) for 30 min. Flow cytometry analysis was done in FACS buffer containing PBS plus 2% FBS using BD LSRFortessa instrument. FlowJo v10.2 software (Treestar) was used for data analysis. Cells were sorted using BD FACSAria.

### ELISA

Five μg/ml peptides (CCP1 (NH2-HQC*HQEST-(cit)-GRSRGRC*GRSGS-COOH (cyclic)), CEP-1 (NH2-C*KIHA-(cit)-EIFDS-(cit)-GNPTVEC*-COOH (cyclic)), Fibrinogen 60-74 (NH2-(cit) PAPPPISGGGY-(cit)-A-(cit)-COOH), or Vimentin 60–75 (NH2-VYAT-(cit)-SSAV-(cit)-L-(cit)-SSVP-COOH) from WuXi AppTec (Beijing, China) in carbonate-bicarbonate buffer (pH 9.6) or recombinant cit-PAD2/4 (produced in house) in PBS (pH 7.4) were coated on ELISA plates (Corning, Corning, USA) overnight at 4°C. All the plates were blocked using 2% BSA (Sigma-Aldrich, Steinheim, Germany) and 5% skimmed milk powder (Sigma-Aldrich) dissolved in PBS. After washing three times with TBST buffer, serial dilutions of sera (1:125 to 1:1000 dilution) were added and incubated for 2 h at room temperature (RT). Goat anti-human IgG-HRP (Yeasen, Shanghai, China) and TMB substrate solution (Beyotime, Shanghai, China) were used for detection. Absorbance was read using R&D plate reader (R&D Systems, Minneapolis, MO) at 450 nm.

### Extraction of RNA and qPCR

TRIzol reagent (Invitrogen, Carlsbad, USA) was used to extract RNA from CD19^+^ B- and CD4^+^ T- cells, as well as CD14^+^ monocytes sorted from clinical samples by flow cytometry. The first-strand cDNA was synthesized using q-PCR kit from Takara Bio (Beijing, China). GAPDH expression was used as control. The forward primers used were listed in **Supplementary Table 2** (ST2). The reverse primers used were from the qPCR detection kit. 2-ΔΔCt method was used to calculate relative RNA expression levels.

### Statistical Analysis

The GraphPad Prism 8, R software version 3.6.3 (The University of Auckland, New Zealand) and SPSS software, version 20.0 (SPSS, Chicago, IL) were used for statistical analysis. Data are given as mean ± SD/SEM and unpaired Student’s t-test was used to compare the data between groups. The p values less than 0.05 was considered as significant at 95% confidence interval.

## Results

### Gene Chip Data

Three microarray data sets for each immune cell, including CD19^+^ B cells (GSE4588, GSE100648 and GSE87095) in RA and (GSE4588, GSE10325 and GSE59250) in SLE, CD4^+^ T cells (GSE4588, GSE56649, and GSE71841) in RA and (GSE4588, GSE103760, and GSE59250) in SLE and CD14^+^ monocytes (GSE71370, GSE38351, and GSE131989) in RA and (GSE4588, GSE103760, and GSE59250) in SLE were obtained from GEO database and used in this study. Detailed information of these data sets is shown in **Table 1**.

**Table 1:** Details for GEO B cells, T cells and Monocytes data in RA and SLE.

Cell types	Diseases	Contributors	Country	Source	Experiment type	GEO	Number of samples	Platform
CD19^+^ B cells	RA	Lauwerys BR. et al. (2006)	Belgium	Peripheral Blood	Expression profiling by array	GSE4588	RA: 7, HC: 9	GPL570
		Thalayasingam N. et al. (2018)	UK	Peripheral Blood	Expression profiling by array	GSE100648	RA: 71, HC: 36	GPL10558
		Julià A. et al. (2016)	Barcelona	Peripheral Blood	Methylation profiling by array	GSE87095	RA:49, HC: 73	GPL13534
	SLE	Lauwerys BR. et al. (2006)	Belgium	Peripheral Blood	Expression profiling by array	GSE4588	SLE: 7, HC: 9	GPL570
		Davis Ll. et al. (2008)	USA	Peripheral Blood	Expression profiling by array	GSE10325	SLE: 14, HC: 9	GPL96
		Absher DM. et al. (2014)	USA	Peripheral Blood	Methylation profiling by array	GSE59250	SLE: 48, HC: 56	GPL13534
CD4^+^ T cells	RA	Lauwerys BR. et al. (2006)	Belgium	Peripheral Blood	Expression profiling by array	GSE4588	RA: 8, HC: 10	GPL570
		Ye H. et al. (2014)	China	Peripheral Blood	Expression profiling by array	GSE56649	RA: 13, HC: 9	GPL570
		Guo S. et al. (2015)	China	Peripheral Blood	Methylation profiling by array	GSE71841	RA: 12, HC: 12	GPL13534
	SLE	Lauwerys BR. et al. (2006)	Belgium	Peripheral Blood	Expression profiling by array	GSE4588	SLE: 8, HC: 10	GPL570
		Lopez CM. et al. (2018)	USA	Peripheral Blood	Expression profiling by array	GSE103760	SLE: 8, HC: 8	GPL17585
		Absher DM. et al. (2014)	USA	Peripheral Blood	Methylation profiling by array	GSE59250	SLE: 78, HC: 71	GPL13534
CD14^+^ Monocytes	RA	Frederiksen KS. et al. (2015)	Denmark	Peripheral Blood	Expression profiling by array	GSE71370	RA: 9, HC: 8	GPL16268
		Smiljanovic B. et al. (2012)	Germany	Peripheral Blood	Expression profiling by array	GSE38351	RA: 8, HC: 12	GPL96
		Barcellos LF. et al. (2019)	USA	Peripheral Blood	Methylation profiling by array	GSE131989	RA: 59, HC: 31	GPL16304
	SLE	Rodriquez-Pla A. et al. (2014)	USA	Peripheral Blood	Expression profiling by array	GSE46907	SLE: 5, HC: 5	GPL96
		Smiljanovic B. et al. (2012)	Germany	Peripheral Blood	Expression profiling by array	GSE38351	SLE: 14, HC: 12	GPL96
		Absher DM. et al. (2014)	USA	Peripheral Blood	Methylation profiling by array	GSE59250	SLE: 27, HC: 28	GPL13534

### Detection of Hub Genes

After analysis using cytohubba of Cytoscape software, top 10 hub genes for each immune cell were identified (**Table 2**). More interestingly, certain common hub genes were found to be present in different cell populations. For example, in RA patients, DICER1 was identified in CD19^+^ B and CD4^+^ T cells, SOCS3 and KRAS were identified in CD4^+^ T cells and CD14^+^ monocytes. Whereas, OAS1, RSAD2, MX1, OAS3, OAS2, and IFIT3 were detected in CD19^+^ B cells and CD14^+^ monocytes from SLE patients.

**Table 2:** Top 10 hub gene of B cells, T cells and monocytes present in RA and SLE.

Cell types	Diseases	Top 10 hub genes
CD19^+^ B cells	RA	BPTF, MRPS28, SF1, PSAP, HNRNPC, ICT1, PHC2, JARID2, DICER1, ERBB2
	SLE	OAS1, XAF1, RSAD2, MX1, STAT1, OASL, IFIT3, OAS3, OAS2, IFIH1
CD4^+^ T cells	RA	STAT5B, SOCS3, JUN, STAT1, KRAS, PTEN, FGFR2, DICER1, ALB, CD44
	SLE	CENPE, MELK, NCAPG, AURKA, BUB1, TOP2A, CCNB1, KIF23, PBK, KIF11
CD14^+^ Monocytes	RA	RPL15, SOCS3, SIRT1, SERBP1, SKP2, TUBA1A, IMP3, EXOSC5, SMAD4, KRAS
	SLE	IFIT1, ISG15, MX1, OAS2, OASL, IFIT2, IFIT3, OAS1, RSAD2, OAS3

### Immune Cells and Autoantibodies Present in the Patient Samples

**Figure 1:**
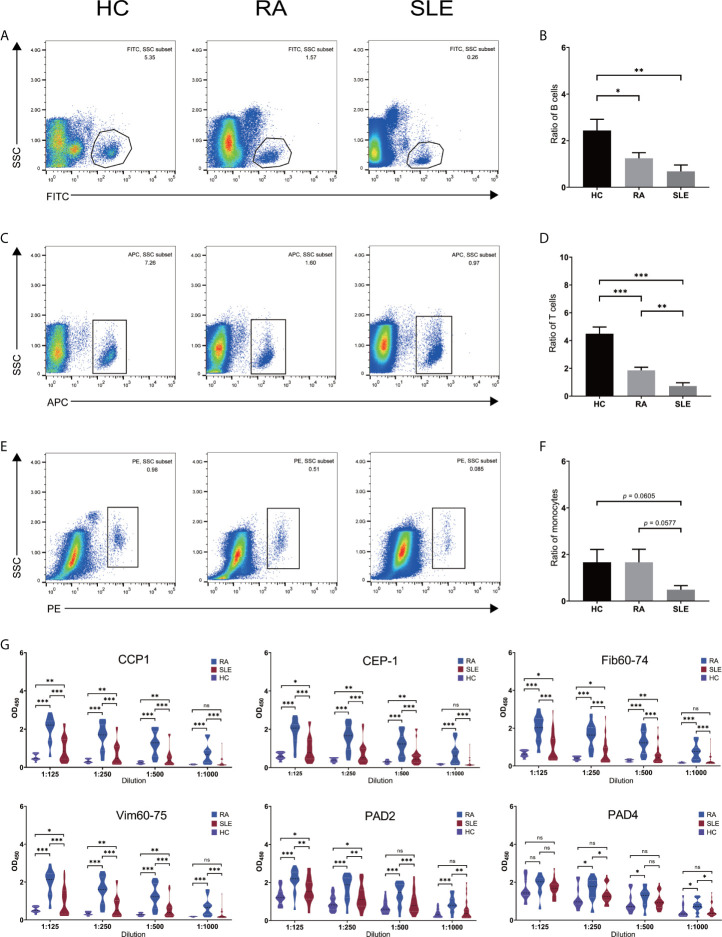
Number of immune cells and level of anti-citrullinated peptide/protein antibodies in the sera of RA, SLE and healthy blood donors. **(A, C, E)** Flow cytometry sorting results for CD19^+^ B- and CD4^+^ T- cells and, CD14^+^ monocytes in HC, RA and SLE. **(B, D, F)** Ratio of CD19^+^ B-, CD4^+^ T- cells and, CD14^+^ monocytes in HC, RA, and SLE. **(G)** The level of anti-CCP1, anti–CEP-1, anti–Fib60-74, anti–Vim60-75, anti-PAD2/4 IgG antibodies in HC, RA, and SLE sera. ns, not significant. *p < 0.05; **p < 0.01; ***p < 0.001.

In order to confirm the validity of identified hub genes, we analyzed the expression of these genes by qPCR using FACS sorted cells. All the FACS sorting results are shown in **Figure 1**. Both CD19^+^ B and CD4^+^ T cells were significantly reduced in both RA and SLE samples, and all the cells in SLE were present relatively less than in RA patients (**Figures 1B, D** **)**. This result is consistent with previously reported results. The total number of peripheral blood B and T cells was reported to be reduced in RA and SLE, probably because of the presence of anti-lymphocyte antibodies (40, 41). In addition, no significant changes in CD14^+^ monocytes were observed in all the three groups (**Figure 1F**). Moreover, the levels of anti–CCP1, anti–CEP-1, anti–Fib60-74, anti–Vim60-75, and anti–cit-PAD2/4 IgG antibodies were increased in both RA and SLE sera, compared to healthy controls (**Figure 1G**). Further clinical laboratory diagnosis data of patients’ samples are given in **Supplementary Table, ST1**.

### Atypically Methylated DEGs in CD19^+^ B Cells of RA and SLE

**Figure 2:**
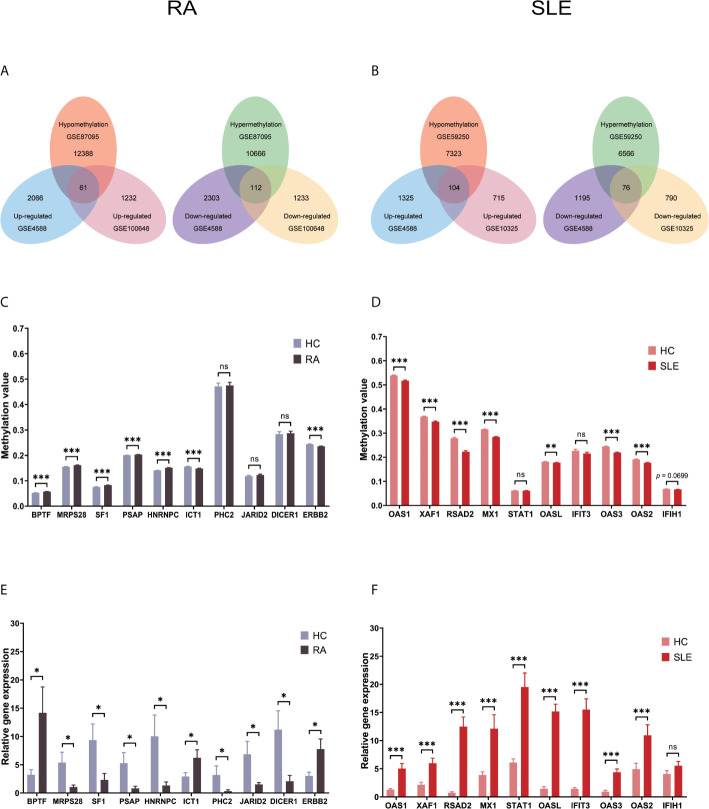
Identification of abnormally methylated DEGs of CD19^+^ B cells in RA and SLE. **(A)** Unusually methylated DEGs of CD19^+^ B cells in RA and **(B)** SLE; **(C)** Methylation level of top 10 hub genes in CD19^+^ B cells from RA and **(D)** SLE; **(E)** Expression levels of top 10 hub genes in CD19^+^ B cells from RA and **(F)** SLE. ns, not significant. *p < 0.05; **p < 0.01; ***p < 0.001.

Each microarray data set was analyzed separately by R package “limma” to filter DEGs or DMGs with the threshold p-value of less than 0.05. After combining DEGs and DMGs expressed in CD19^+^ B cells, 61 hypomethylated-highly expressed genes and 112 hypermethylated-low expressed genes were acquired by overlapping three microarrays (GSE4588, GSE100648 and GSE87095) in RA (**Figure 2A**). On the other hand, 104 hypomethylated-highly expressed genes and 76 hypermethylated-low expressed genes were obtained by overlapping three microarrays (GSE4588, GSE10325, and GSE59250) in SLE (**Figure 2B**). To identify top 10 hub genes, DiseaseMeth version 2.0 analysis was used. In CD19^+^ B cells, the mean methylation levels of BPTF, MRPS28, SF1, PSAP, and HNRNPC were significantly higher, while ICT1 and ERBB2 methylation levels were lower in RA compared to healthy controls (**Figure 2C**). In the case of SLE, compared to healthy individuals, the mean methylation levels of OAS1, XAF1, RSAD2, MX1, OASL, OAS3, and OAS2 were lower (**Figure 2D**), while the methylation levels of other three genes were not significant. The expression of top 10 hub genes was confirmed by qPCR using FACS sorted immune cells. BPTF, ICT1, and ERBB2 were significantly expressed at higher levels, while MRPS28, SF1, PSAP, HNRNPC, PHC2, JARID2, and DICER1 were expressed significantly lower in RA (**Figure 2E**). OAS1, XAF1, RSAD2, MX1, STAT1, OASL, IFIT3, OAS3, and OAS2 but not IFIH1 were significantly expressed at higher levels in SLE (**Figure 2F**).

**Figure 3:**
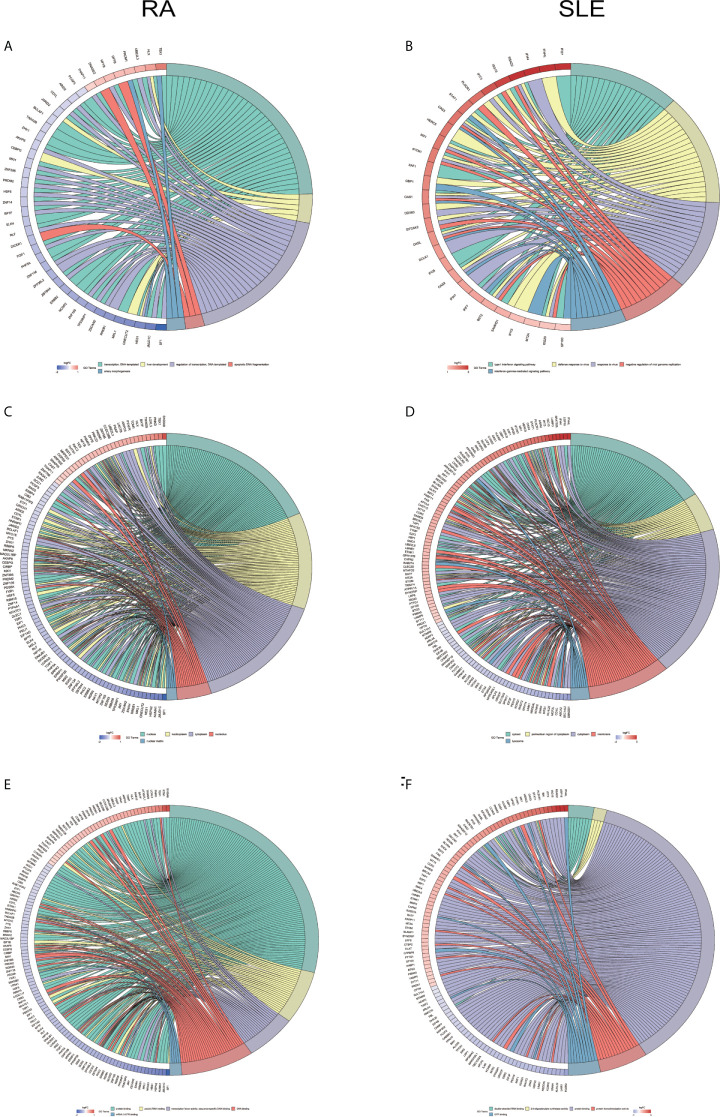
Chord plot depicting the relationship between aberrantly methylated DEGs of CD19^+^ B cells between RA and SLE. **(A)** Top 5 GO terms of biological process in RA; and **(B)** SLE; **(C)** Top 5 GO terms of cellular components in RA and **(D)** SLE; **(E)** Top 5 GO terms of molecular functions in RA and **(F)** SLE. GO, Gene Ontology.

All the differentially expressed and methylated genes from GO enrichment analysis for CD19^+^ B cells are shown in the **supplementary Files**, **SF1A** and **SF1B**, for RA and SLE, respectively. Firstly, in the CD19^+^ B cells from RA, we detected enrichment of aberrantly methylated DEGs related to “transcription, DNA-templated,” “regulation of transcription, DNA-templated,” “apoptotic DNA fragmentation,” “artery morphogenesis,” and “liver development” for biological processes in terms of GO (**Figure 3A**). For cellular component, “nucleus,” “nucleoplasm,” “cytoplasm,” “nucleolus,” and “nuclear matrix” were the most significantly enriched terms (**Figure 3C**). In addition, few molecular functional terms, like “protein binding,” “DNA binding,” sequence-specific DNA binding,” “poly(A) RNA binding,” “mRNA 3’-UTR binding,” and “transcription factor activity,” were found to be enriched (**Figure 3E**). Secondly, the aberrantly methylated DEGs enrichment in SLE were found to be connected to “type I interferon signaling pathway,” “interferon-gamma-mediated signaling pathway,” “response to virus,” “defense response to virus,” and “negative regulation of viral genome replication” GO terms for biological processes (**Figure 3B**). For cellular components, “cytosol,” “perinuclear region of cytoplasm,” “cytoplasm,” “membrane,” and “lysosome” were the most significantly enriched terms (**Figure 3D**). In addition, few molecular function terms, such as “double-stranded RNA binding,” “2′-5′-oligoadenylate synthetase activity,” “protein binding,” “protein homodimerization activity,” and “GTP binding” were enriched for SLE (**Figure 3F**). The top five significant GO terms from DAVID were presented in **Supplementary Table, ST3**.

**Figure 4:**
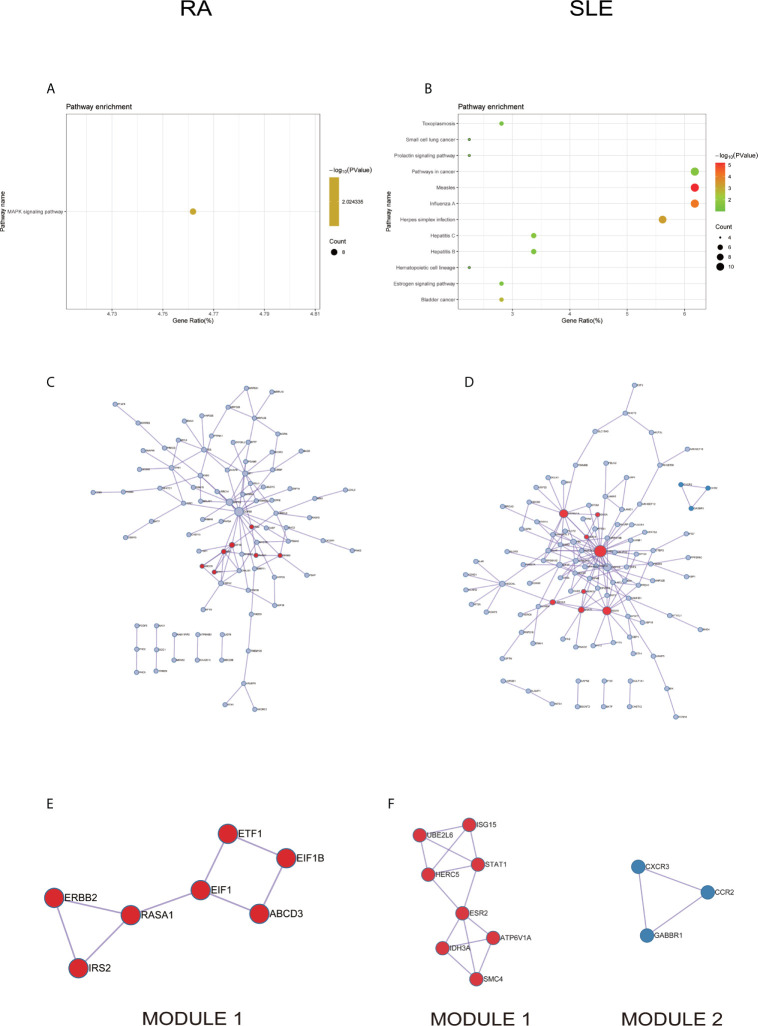
The KEGG pathway analysis, PPI network and the most significant modules between RA and SLE in CD19^+^ B cells. **(A, B)** The KEGG pathway analysis of RA and SLE; **(C, D)** PPI network of CD19^+^ B cells analyzed by String software between RA and SLE; **(E, F)** The significant modules of CD19^+^ B cells identified by MCODE between RA and SLE. DEG, differentially expressed gene; PPI, protein-protein interaction.

In KEGG analysis of irregularly methylated DEGs of CD19^+^ B cells, enrichment of mitogen-activated protein kinase (MAPK) and measles signaling pathways were noted in RA and SLE, respectively (**Figures 4A, B** **)**. All the KEGG pathway enrichment analyses of CD19^+^ B cells were shown in **Supplementary Table, ST4**. PPI network for CD19^+^ B cells was presented in **Figures 4C, D** for RA and SLE and all the modules were displayed in **Figures 4E, F**. The functional analysis of PPI network for aberrantly methylated DEGs was given in **Supplementary Table, ST5** and all the module analyses were presented in **Supplementary Table, ST6**.

### Identification of Anomalously Methylated-DEGs Between RA and SLE in CD4^+^ T Cells

**Figure 5:**
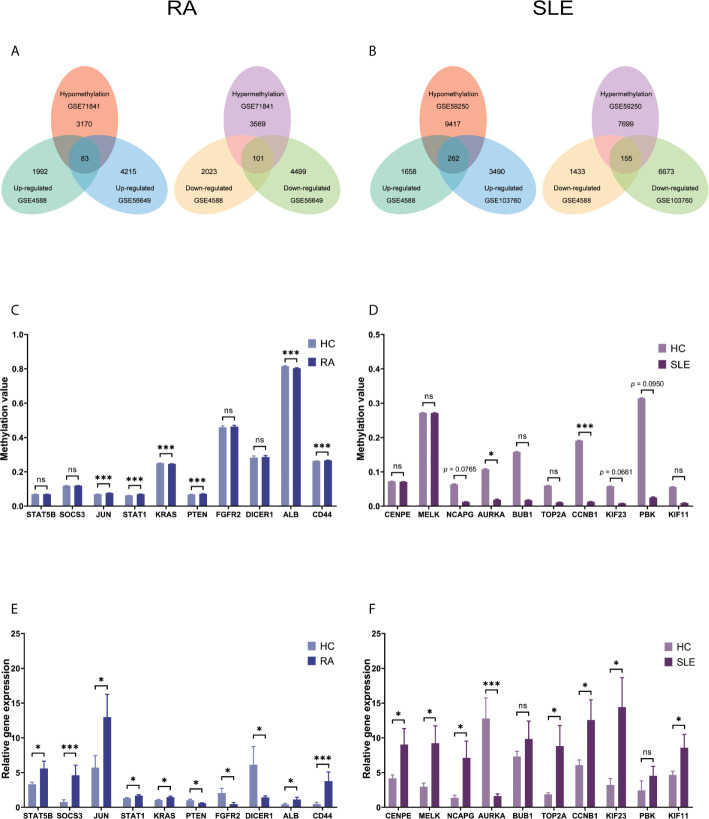
Identification of aberrantly methylated DEGs of CD4^+^ T cells in RA and SLE. **(A)** Aberrantly methylated DEGs of CD4^+^ T cells in RA and **(B)** SLE; **(C)** Methylation level of top 10 hub genes in CD4^+^ T cells from RA; **(D)** and SLE; **(E)** Expression levels of top 10 hub genes in CD4^+^ T cells from RA and **(F)** SLE. ns, not significant. *p < 0.05; ***p < 0.001.

After combining DEGs and DMGs expressed in CD4^+^ T cells, 83 hypomethylated-highly expressed genes and 101 hypermethylated-low expressed genes were identified by overlapping three microarrays (GSE4588, GSE56649, and GSE71841) in RA (**Figure 5A**). Similarly, 262 hypomethylated-highly expressed genes and 155 hypermethylated-low expressed genes were identified by overlapping three microarrays (GSE4588, GSE103760, and GSE59250) in SLE (**Figure 5B**). To identify top 10 hub genes, DiseaseMeth version 2.0 analysis for CD4^+^ T cells was used. Significantly higher mean methylation levels of JUN, STAT1, PTEN, and CD44 genes, and lower methylation levels of KRAS and ALB genes were detected in RA compared to healthy samples (**Figure 5C**). Whereas, significantly lower mean methylation levels of AURKA and CCNB1 were identified in SLE compared to healthy controls (**Figure 5D**), while methylation level of other genes were insignificant. Expression level of top 10 hub genes was detected by qPCR. Significantly high-level expression of STAT5B, SOCS3, JUN, STAT1, KRAS, ALB, and CD44 were observed, whereas, lower expression of PTEN, FGFR2 and DICER1 were noticed in RA (**Figure 5E**). In SLE, higher level expression of CENPE, MELK, NCAPG, TOP2A, CCNB1, KIF23, and KIF11 genes and low-level expression of AURKA were discovered (**Figure 5F**).

**Figure 6:**
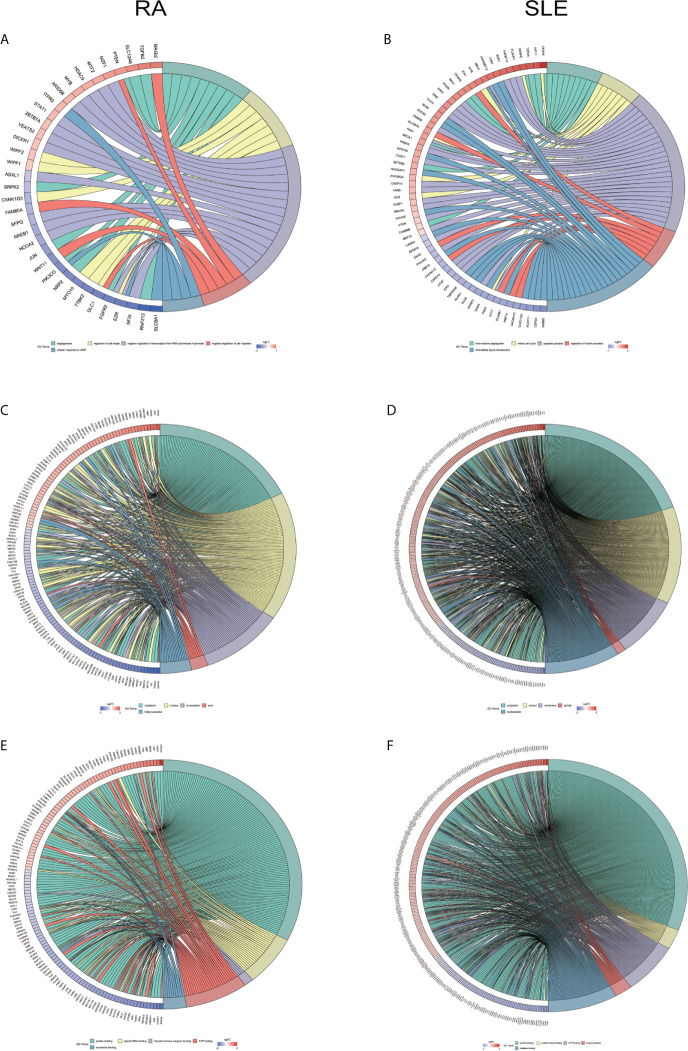
Chord plot depicting the relationship between aberrantly methylated DEGs of CD4^+^ T cells between RA and SLE. **(A)** Top 5 GO terms of biological process in RA and **(B)** SLE; **(C)** Top 5 GO terms of cellular components in RA and **(D)** SLE; **(E)** Top 5 GO terms of molecular functions in RA and **(F)** SLE. GO, gene ontology.

**Figure 7:**
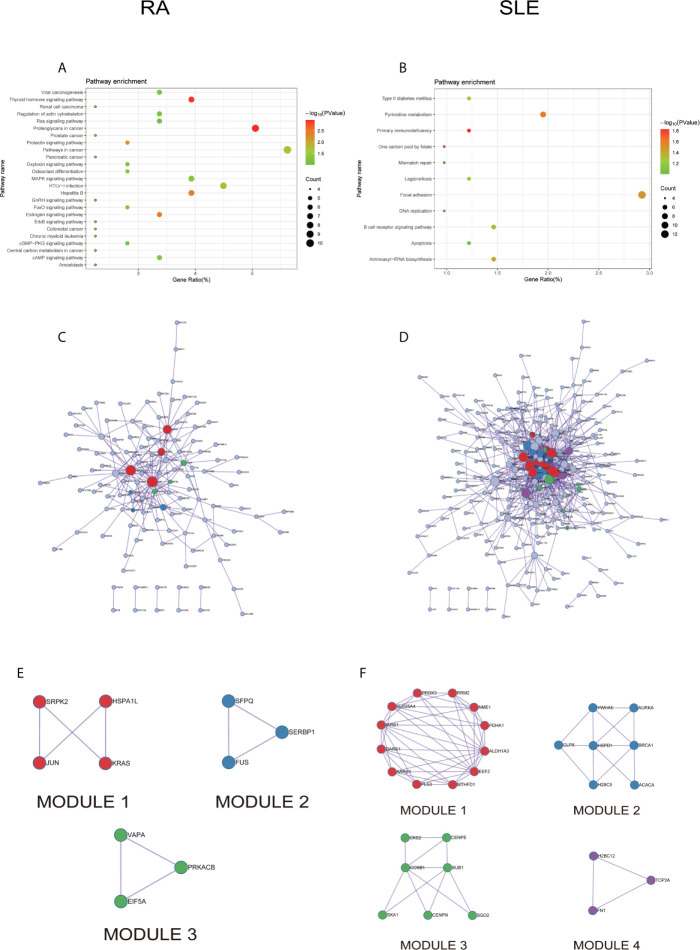
The KEGG pathway analysis, PPI network and the most significant modules between RA and SLE in CD4^+^ T cells. **(A, B)** KEGG pathway analysis of RA and SLE; **(C, D)** PPI network of CD4^+^ T cells analyzed by String software between RA and SLE; **(E, F)** The significant modules of CD4^+^ T cells identified by MCODE between RA and SLE. DEG: differentially expressed gene; PPI: protein-protein interaction.

All the differentially expressed and methylated genes from GO enrichment analysis for CD4^+^ T cells were shown in **Supplementary Figures**, **SF2A** and **SF2B** for RA and SLE. Firstly, for the CD4^+^ T cells in RA, we detected the aberrantly methylated DEGs enrichment related to “angiogenesis,” “regulation of cell shape,” “negative regulation of transcription from RNA polymerase II promoter,” “negative regulation of cell migration,” and “cellular response to cAMP” GO terms for biological processes (**Figure 6A**). In terms of cellular components, “cytoplasm,” “nucleus,” “nucleoplasm,” “axon,” and “Golgi apparatus” were the most significantly enriched terms (**Figure 6C**). In addition, few molecular function terms, such as “protein binding,” “poly(A) RNA binding,” “ATP binding,” “nucleotide binding,” and “thyroid hormone receptor binding,” were enriched (**Figure 6E**). Secondly, enrichment of the GO terms for aberrantly methylated DEGs in SLE such as “chromosome segregation,” “mitotic cell cycle,” “apoptotic process,” “regulation of insulin secretion,” and “intracellular signal transduction” were most significant in biological processes (**Figure 6B**). In the cellular components, “cytoplasm,” “cytosol,” “membrane,” “spindle,” and “nucleoplasm” were the most significantly enriched terms (**Figure 6D**). In addition, few molecular functional terms, such as “protein binding,” “enzyme binding,” “protein kinase binding,” “ATP binding,” and “metal-ion binding” were enriched for SLE (**Figure 6F**). The top five significant GO terms from DAVID were given in **Supplementary Table, ST3**. In KEGG pathway analysis of aberrantly methylated DEGs of CD4^+^ T cells, proteoglycans in cancer and primary immunodeficiency signaling pathways were significantly enriched for RA and SLE (**Figures 7A, B** **)**, respectively. All the KEGG pathway enrichment analyses of CD4^+^ T cells were shown in **Supplementary Table, ST4**. PPI network for CD4^+^ T cells was shown in **Figures 7C, D** for RA and SLE, respectively, and all the modules were displayed in **Figures 7E, F**. The functional analysis of PPI network for unusually methylated DEGs was given in **Supplementary Table 5 (ST5)**, and all the module analyses were shown in **Supplementary Table 6 (ST6)**.

### Exceptionally Methylated-DEGs Between RA and SLE in CD14^+^ Monocytes

**Figure 8:**
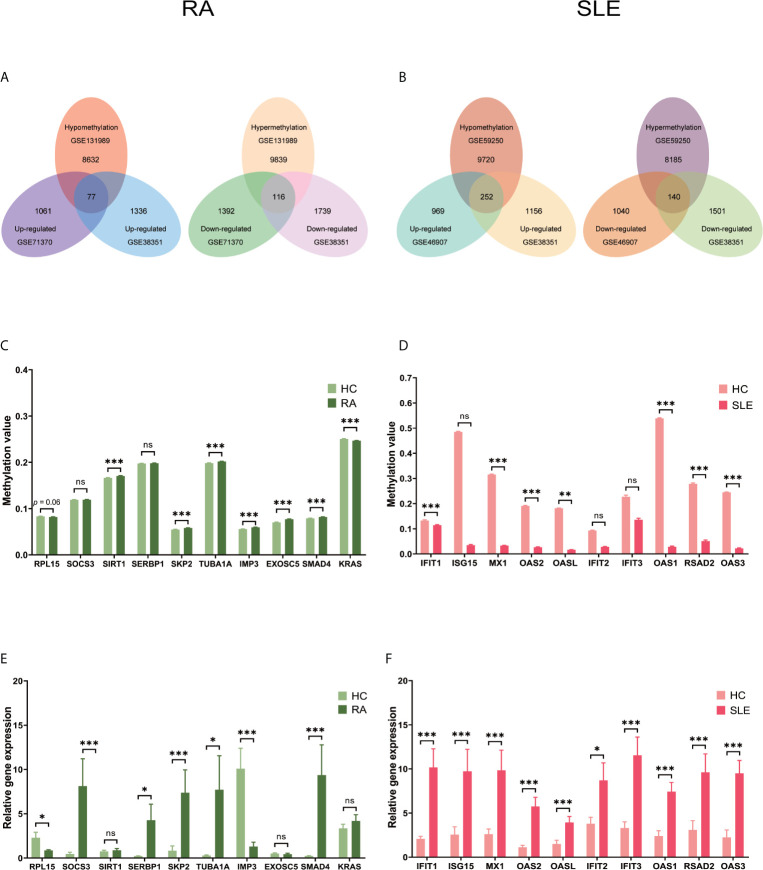
Identification of aberrantly methylated DEGs of CD14^+^ monocytes in RA and SLE. **(A)** Aberrantly methylated DEGs of CD14^+^ monocytes in RA and **(B)** SLE; **(C)** Methylation level of top 10 hub genes in CD14^+^ monocytes from RA and **(D)** SLE; **(E)** Expression levels of top 10 hub genes in CD14^+^ Monocytes from RA and **(F)** SLE. ns, not significant. *p < 0.05; **p < 0.01; ***p < 0.001.

After combining DEGs and DMGs expressed in CD14^+^ monocytes, 77 hypomethylated-highly expressed genes and 116 hypermethylated-low expressed genes were identified by overlapping three microarrays (GSE71370, GSE38351, and GSE131989) in RA (**Figure 8A**), whereas 252 hypomethylated-highly expressed genes and 140 hypermethylated-low expressed genes were identified by overlapping three microarrays (GSE46907, GSE38351, and GSE59250) in SLE (**Figure 8B**). To identify top 10 hub genes in CD14^+^ monocytes, DiseaseMeth version 2.0 analysis was performed, which showed significantly higher mean methylation levels for SIRT1, SKP2, TUBA1A, IMP3, EXOSC5, and SMAD4, while KRAS had significantly lower methylation level in samples from RA patients compared to healthy individuals (**Figure 8C**). Similarly, IFIT1, MX1, OAS2, OASL, OAS1, RSAD2, and OAS3 had significantly lower mean methylation levels in SLE compared to healthy controls (**Figure 8D**), while for other three genes insignificant differences in methylation level were observed. Expression levels of the top 10 hub genes were identified by qPCR. In RA, significantly high-level expression of SERBP, SKP2, TUBA1A, SMAD4, and SOCS3 were observed, while low-level expression of RPL15 and IMP3 were found (**Figure 8E**). On the other hand, all the top 10 hub genes IFIT1, ISG15, MX1, OAS2, OASL, IFIT2, IFIT3, OAS1, RSAD2, and OAS3 were highly expressed in SLE (**Figure 8F**).

**Figure 9:**
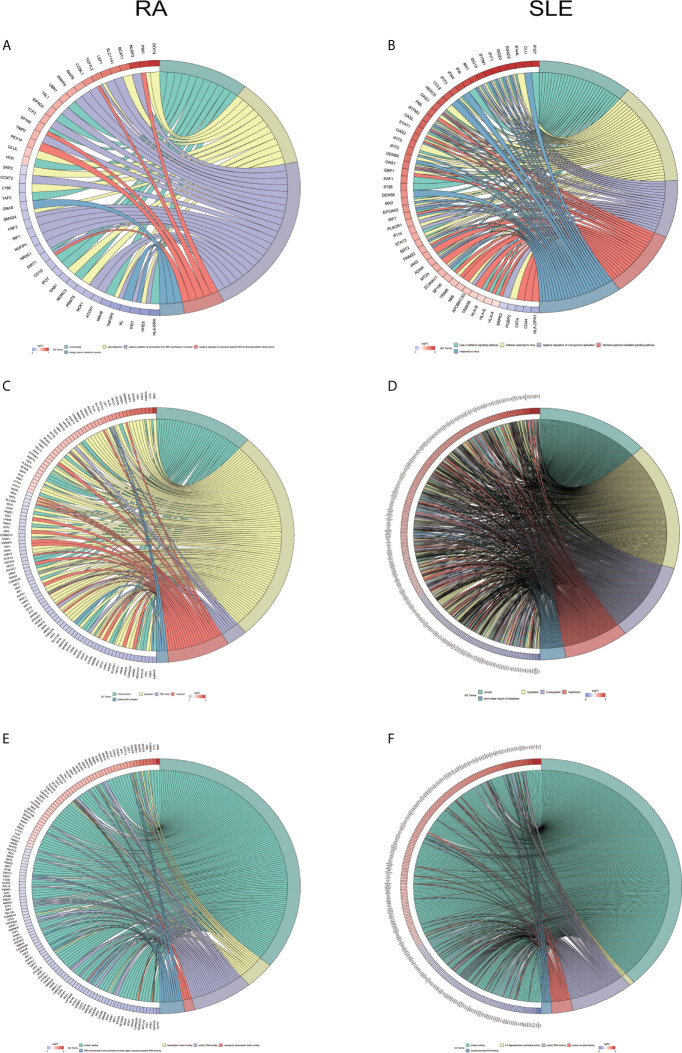
Chord plot showing a relationship between aberrantly methylated DEGs of CD14^+^ monocytes between RA and SLE. **(A)** Top 5 GO terms of biological process in RA and **(B)** SLE; **(C)** Top 5 GO terms of cellular components in RA and **(D)** SLE; **(E)** Top 5 GO terms of molecular functions in RA and **(F)** SLE. GO, Gene Ontology.

**Figure 10:**
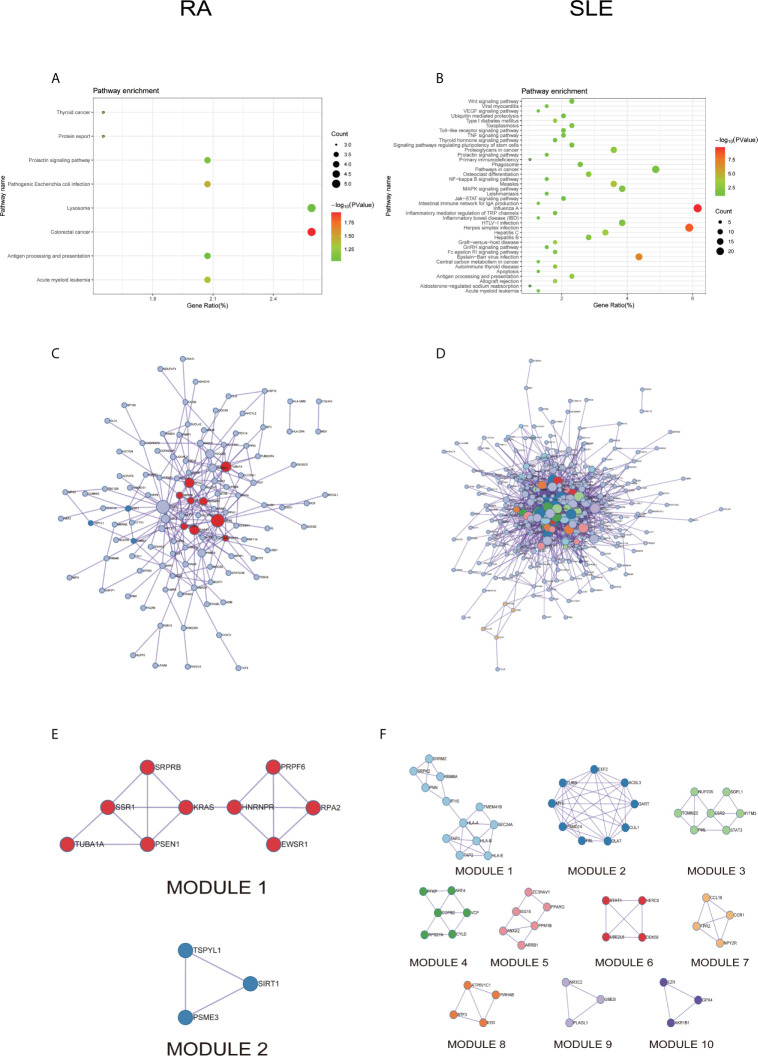
The KEGG pathway analysis, PPI network and the most significant modules between RA and SLE in CD14^+^ monocytes. **(A, B)** The KEGG pathway analysis of RA and SLE; **(C, D)** The PPI network of CD14^+^ monocytes was analyzed by String software between RA and SLE; **(E, F)** The significant modules of CD14^+^ monocytes were identified by MCODE between RA and SLE. DEG, differentially expressed gene; PPI, protein-protein interaction.

All the differentially expressed and methylated genes from GO enrichment analysis for CD14^+^ monocytes were presented in supplementary figures, SF3A and SF3B for RA and SLE, respectively. Firstly, for the CD14^+^ monocytes in RA, we detected enrichment of abnormally methylated DEGs like “viral process,” “cell proliferation,” “positive regulation of transcription from RNA polymerase II promoter,” “energy reserve metabolic process,” and “negative regulation of sequence-specific DNA binding transcription factor activity” GO terms in biological processes (**Figure 9A**). In the cellular components, “mitochondrion,” “cytoplasm,” “PML body,” “nucleolus,” and “protein-DNA complex” were the most significantly enriched terms (**Figure 9C**). In addition, few molecular function related terms, such as “protein binding,” “poly(A) RNA binding,” “transcription factor binding,” “repressing transcription factor binding,” and “RNA polymerase II core promoter proximal region sequence-specific DNA binding” were enriched (**Figure 9E**). Secondly, significant enrichment of GO terms in SLE for the unusually methylated DEGs are “type I interferon signaling pathway,” “interferon-gamma-mediated signaling pathway,” “defense response to virus,” “response to virus,” and “negative regulation of viral genome replication” for biological processes (**Figure 9B**). In the cellular components, “cytosol,” “cytoplasm,” “nucleoplasm,” “membrane,” and “perinuclear region of cytoplasm” were the most significantly enriched terms (**Figure 9D**). Furthermore, few molecular functional terms, such as “protein binding,” “2′-5′-oligoadenylate synthetase activity,” “poly(A) RNA binding,” “protein complex binding,” and “double-stranded RNA binding” were enriched for SLE (**Figure 9F**). The top five GO terms enriched in DAVID were presented in **Supplementary Table 3 (ST3)**. In KEGG pathway enrichment of abnormally methylated DEGs in CD14^+^ monocytes, colorectal cancer and Influenza A signaling pathways were significantly enriched in RA and SLE, respectively (**Figures 10A, B** **)**. All the KEGG pathway enrichment analysis of CD14^+^ monocytes were shown in **Supplementary Table 4 (ST4)**.

PPI network for CD14^+^ monocytes was shown in **Figures 10C, D** for RA and SLE, respectively. All the modules were displayed in **Figures 10E, F**. The functional analysis of PPI network for unusually methylated DEGs was given in **Supplementary Table 5 (ST5)**, and all the module analyses were shown in **Supplementary Table 6 (ST6)**.

### Evaluation of Hub Genes

**Figure 11:**
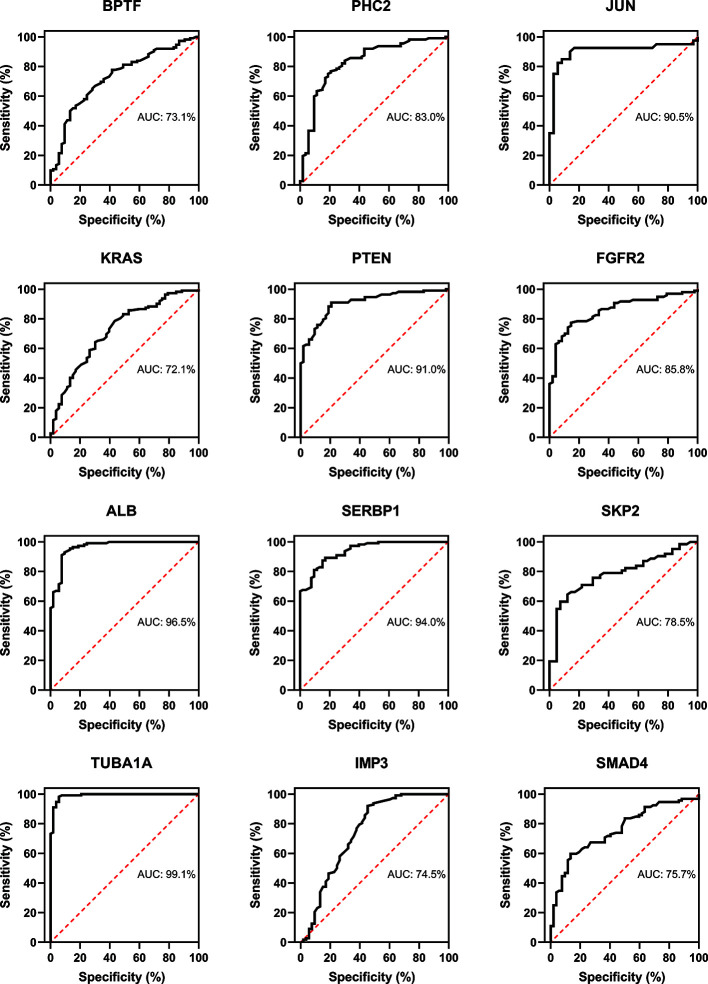
Confirmation of RA hub genes by ROC analysis. Out of 30 genes of immune cells filtered by MCODE, 12 genes having AUC > 0.70 were identified as biomarker genes for RA.

**Figure 12:**
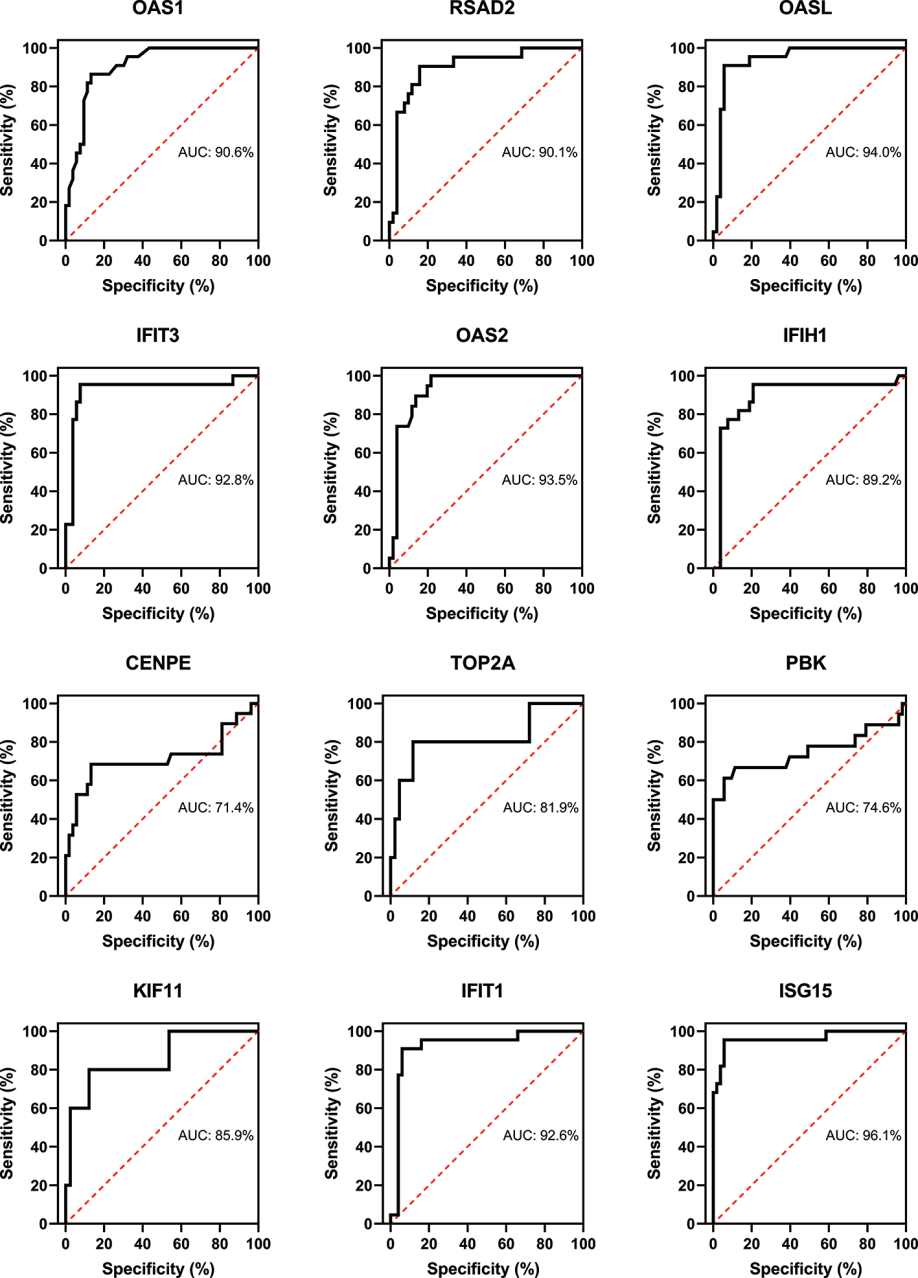
Confirmation of SLE hub genes by ROC analysis. Out of 30 genes of immune cells filtered by MCODE, 12 genes having AUC > 0.70 were identified as biomarker genes for SLE.

Three immune cells and 30 hub genes filtered by MCODE in total were used to differentiate RA patients from healthy individuals, and thus deemed as biomarker genes. To substantiate the expression of these 30 genes in blood cells from other patients, we have chosen GSE17755 data set and did ROC analysis using R Studio. Among the 30 hub genes, 12 genes (BPTF, PHC2, JUN, KRAS, PTEN, FGFR2, ALB, SERB-1, SKP2, TUBA1A, IMP3, and SMAD4), which had AUC above 0.70 were appraised as biomarker genes, because they can distinguish RA patients in a more specific and sensitive manner (**Figure 11**). Similarly, among the 30 hub genes in SLE, 12 genes (OAS1, RSAD2, OASL, IFIT3, OAS2, IFIH1, CENPE, TOP2A, PBK, KIF11, IFIT1, and ISG15), which had AUC above 0.70 were appraised as biomarker genes, because they can be used to specifically diagnose SLE patients with high sensitivity (**Figure 12**). Except for the ROC values of CENPE (p = 0.006), TOP2A (p = 0.0208), PBK (p = 0.0019), and KIF11 (p = 0.0095) genes expressed in the T cells of SLE, all the other genes in SLE and RA had p values < 0.0001.

## Discussion

Understanding various pathogenic mechanisms present in immune cells of RA and SLE is indispensable to develop better diagnosis and treatment strategies. We did a combined analysis of epigenetics and genomics data, to dissect the abnormally methylated DEGs present in the immune cells from RA and SLE. Presence of exceptionally altered methylome of DEGs (173 and 180 in CD19^+^ B cells, 184 and 417 in CD4^+^ T cells and 193 and 392 in CD14^+^ monocytes) from RA and SLE patients were identified using several bioinformatics tools. Understanding the pattern of expression of these enriched genes and their abnormal methylation signature might contribute to identify new biomarkers and confirm pathways operating in these autoimmune diseases at cellular level that could facilitate development of specific targeted therapies.

In CD19^+^ B cells, as revealed by DAVID analysis, aberrantly methylated DEGs were enriched in the biological processes of DNA templated transcription and its regulation. In RA, both central and peripheral B cell tolerance mechanisms are compromised, resulting in an increased number of autoreactive B cells (42) leading to an enhanced autoantibody synthesis and cellular metabolism (43). On the other hand, signaling cascades involving type I interferon and anti-viral immune responses were enriched in SLE. It is well known that lupus pathogenesis also involves the interferon pathway and 2’5’-oligoadenylate synthetase (OAS) (44, 45). This pinpoints to the prevailing differences in B cell functions contributing to RA and SLE disease processes, though autoantibodies have a major contribution to disease initiation and development in both these diseases. In CD4^+^ T cells, exceptionally methylated DEGs were enriched in the biological processes of angiogenesis, DNA-templated transcription, transport, and regulation of cell shape in RA. This indicates a prominent feature of biological functions of T cells in RA (46). An association between RA and HLA-DRB1 is the clear evidence available so far that strongly supports a pathogenic role for CD4^+^ T cells in disease initiation and perpetuation. In addition, RA associated allelic variants of several genes like PTPN22, CTLA4, ZAP70, and PADI4 encode molecules that are directly involved in T-cell activation pathways. Synovial biopsies provide a convincing evidence for the contribution of T cells in the chronic immune activation pathways operating in RA patients (47). In SLE, CD4^+^ T cells are mainly enriched in association with apoptosis. T cells are also quite active in SLE, a disorder mainly caused by defects in apoptosis leading to amplification of inflammatory responses. In CD14^+^ monocytes, aberrantly methylated DEGs were enriched in biological processes related to DNA-templated transcription and positively regulated RNA polymerase II phosphorylation in RA. CD14^+^ monocytes are mainly located in the capillaries and small arteries and veins, patrolling near vascular endothelial cells (48). In RA, peripheral blood CD14^+^ monocytes pass through vascular endothelial cells into tissues and get differentiated into macrophages that can secrete several pro-inflammatory factors. In addition, monocytes can also differentiate into dendritic cells (DCs) with a strong antigen presenting ability (49). Therefore, its protein expression and transcription levels are associated with high metabolic rate. Interestingly, decreased ser/thr kinase Akt pathway activation in monocytes can contribute to the perpetuation of systemic inflammation in RA (50). In SLE, type I interferon signaling pathway, defense response to virus and OAS activity were mainly enriched in CD14^+^ monocytes, similar to B cells, because virus infection and interferon pathway are closely related to the pathogenesis of SLE.

**Figure 13:**
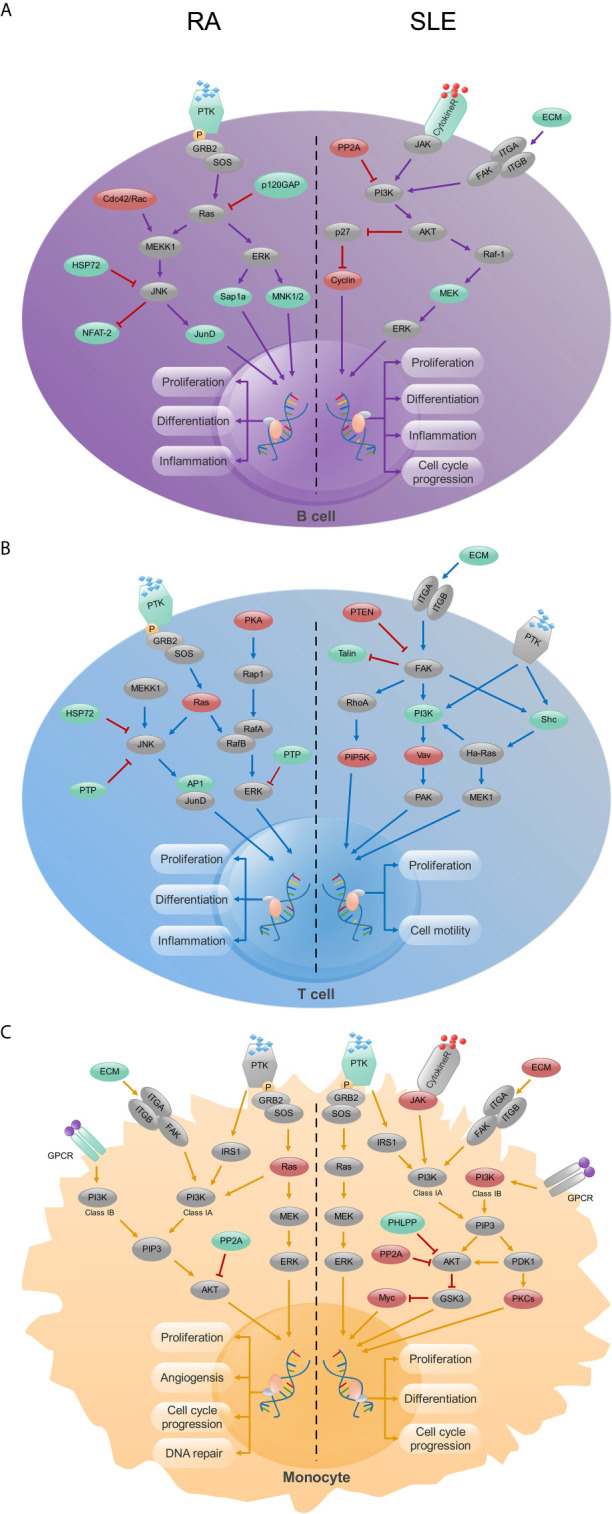
The KEGG pathway analysis of CD19^+^ B-, CD4^+^ T- cells and CD14^+^ monocytes in RA and SLE. **(A)** In CD19^+^ B cells, MAPK and PI3K signaling pathways were enriched in RA and SLE, respectively. **(B)** In CD4^+^ T cells, MAPK and focal adhesion signaling pathways were enriched in RA and SLE, respectively. **(C)** PI3K signaling pathway was enriched in CD14^+^ monocytes from both RA and SLE. Up-regulated, down-regulated and unchanged genes are marked in red, green and gray colors, respectively.

During initial stages of autoimmune disease development, due to the decreased tolerance of immune cells (B cells and T cells), activation of inflammatory factors and production of autoantibodies, immune cells will be damaged and the related inflammatory pathways, such as MAPK or phosphatidylionsitol 3-kinase (PI3K) will get activated (51). KEGG analysis demonstrated a significant enrichment of MAPK and measles pathways in CD19^+^ B cells from RA and SLE, respectively. This clearly suggests functional difference of B cells operating in both RA and SLE. Thyroid hormone and estrogen signaling, hepatitis B and MAPK pathways were enriched in CD4^+^ T cells from RA, while primary immunodeficiency pathway was significantly enriched in SLE. Recently, an enhanced prevalence and risk for thyroid dysfunctions, especially overt hypothyroidism, in arthritis patients were reported (52). Similarly, RA patients have an increased risk for HBV infection than non-RA subjects (53). Moreover, estrogen also modulates T-cell intrinsic signaling pathways in RA (54). This observation is also consistent with the frequent dysregulation in the MAPK signaling pathway observed in RA patients (55). Interestingly, the MAPK pathway is enriched in both CD19^+^ B- and CD4^+^ T-cells. However, we need more experiments to ascertain whether the MAPK pathway is a common regulatory pathway in both B cells and T cells, or operating in other immune cells as well. Through “Pathview” analysis of aberrantly methylated DEGs, we found certain connection between the pathways operating in RA and SLE (**Supplementary Figure, SF4**). In CD19^+^ B- and CD4^+^ T-cells, MAPK and PI3K pathways were enriched in RA and SLE, respectively (**Figures 13A, B** **)**. More interestingly, enrichment of PI3K pathway was found in CD14^+^ monocytes in both of these autoimmune diseases (**Figure 13C**). In this context, it is of interest to note that for therapeutic interventions, MAPK regulators are increasingly being tested as inflammatory as well as immune modulators (56). Similarly, both in humans and animals affected by lupus, activity of PI3K was found to be increased, and PI3K inhibitors have attenuated lupus inflammation and disease manifestations (57). This demonstrates prevailing clear differences in RA and SLE pathogenesis at cellular level. However, in monocytes, PI3K pathway is enriched in both these diseases, suggesting existence of common pathways as well, which should be considered, while prescribing drugs and designing treatments for these patients (58) as well as during developmental stages of new drugs (**Supplementary Table, ST7**).

PPI network and module analysis in CD19^+^ B cells from RA and SLE suggested B cells in RA patients might be mainly related to the production of pathogenic autoantibodies, while in SLE patients, though autoantibody mediated disease manifestations are quite prominent, they are also associated with the activation of interferon pathway, which is consistent with our GO analysis. In CD4^+^ T cells, RA but not SLE is related to the estrogen signaling pathway. Fas and interferon signaling pathways were enriched in CD14^+^ monocytes from RA and SLE, respectively. Our findings highlight the significance of regulation in these essential biological pathways by aberrant methylation, which warrant further investigation. PPI network of abnormally methylated DEGs demonstrates an overview of their functional interactions. Among them, top 10 hub genes from each immune cell were selected both in RA and SLE. Using methylation database and qPCR, we confirmed the relation between top 10 hub genes in each immune cell. Validation of RA and SLE candidate hub genes by ROC analysis showed 12 genes (BPTF, PHC2, JUN, KRAS, PTEN, FGFR2, ALB, SERB-1, SKP2, TUBA1A, IMP3, and SMAD4) for RA and 12 genes (OAS1, RSAD2, OASL, IFIT3, OAS2, IFIH1, CENPE, TOP2A, PBK, KIF11, IFIT1, and ISG15) for SLE could be considered as biomarker genes, for diagnosis. We have used data from blood cells of RA and SLE patients as well as healthy controls from another data set GSE17755 for validating the hub genes by ROC analysis using RStudio. However, all the data sets used in our study were coming from GEO database only. This is because other databases available as of now (TCGA, GEPIA, ICGC, and Oncomine) focus mainly on samples from cancer patients. So, whether the proposed genes can be used for early diagnosis or screening of general population for risk individuals involving these diseases, along with other well-known genetic and environmental factors remains to be investigated.

As we have mentioned earlier, MAPK and PI3K signaling pathways play a crucial role in the RA development that was further substantiated by functional studies attributed to some of the identified hub genes in this study. Among the potential biomarker genes, in RA patients, deletion of BPTF specifically in Treg cells decreased the expression of Foxp3 and increased the infiltration of lymphocytes in different organs causing systemic autoimmunity, showing the importance of this gene in T cell function and immune homeostasis (59). Knockout of the BPTF gene down-regulated the expression of phosphorylated Erk1/2, PI3K, and Akt proteins, and induced the cleavage of caspase-8, caspase-7, and PARP proteins, thereby inhibited MAPK and PI3K/AKT signal transduction and activation pathways (60). Though BPTF is crucial in T cell development, its role in B cells is yet to be clarified. In our analysis, initiation of RA seems to be closely related to the activation of the MAPK pathway, so the expression and activation of the BPTF gene may be used as a specific marker for the activation of MAPK pathway. Besides, another proposed gene JUN encodes C-Jun that can activate the MAPK pathway and regulate the expression of inflammatory response-related genes, and affect cell proliferation and cell cycle as well (61). Similarly, the KRAS gene encoding the K-Ras protein is part of the RAS/MAPK pathway. Another gene, PTEN is the main contributor to Treg function and for differential regulation of Th17 functions (62) and PTEN deficiency inhibited MAPK signal transduction pathway and the expression of inflammatory cytokines IL-12/23, IL-6, and TNF-α (63). FGFR2 signaling activates multiple downstream pathways, including RAS/MAPK and PI3K/AKT pathways (64). SKP2 is associated with the activation of the AKT/mTOR and Ras/MAPK pathways (65). IMP3 activates IGF-2, thereby involved in the regulation and activation of PI3K and MAPK pathway (66), whereas SMAD4 is the mediator of the TGFβ signaling pathway, which can regulate the activation of the MAPK pathway (67). Hence, appropriate use of MAPK and PI3K inhibitors can inhibit immune cell-related pro-inflammatory pathways operating in RA and reverse the ongoing inflammatory responses (68). Similar to RA, the activation of PI3K pathway is significantly important in SLE patients, and the therapeutic drugs targeting PI3K pathway was shown to be useful in SLE (69). Earlier reports also showed a correlation between the expression levels of OAS1, OASL, ISG15, IFIH1, IFIT1, and OAS2 genes and disease activity and/or clinical symptoms in SLE patients (70, 71). Both JAK3 and PI3K inhibitors inhibited the expression of ISG15 and OAS1 (72). Similarly, IFIT1 and RSAD2 also have a critical function in lupus development (73). IFIT3 can be targeted to block type I IFN and inflammatory cytokine synthesis by cGAS/STING pathway in lupus affected individuals (74). Most importantly, over expression of IFIT1 or IFIT3 increased EGFR and AKT expression, which is closely related to the activation of the PI3K pathway (75). TOP2A can also activate the PI3K/AKT signaling pathway leading to cell migration (76), whereas an increased expression of KIF11 inhibited the activation of PI3K/AKT signaling pathway (77). In summary, the potential biomarker genes proposed for SLE diagnosis are closely related to PI3K pathway, and the use of PI3K inhibitors reversed the changes associated with ISG15, OAS1, OASL, and IFIT1 (72, 78, 79). However, not all the identified biomarker genes are well connected yet with RA and SLE disease process, which also needs further research.

Many labs currently focus on the changes present in gene methylation profiles, especially in lymphocytes. CD4^+^ T cells from SLE patients have hypomethylation in the promoter and enhancer regions of ITGAL and TNFSF7 (80, 81). Similarly, the changes in the methylation status of CD40L, FOXP3, RFX1, and CREM led to changes in the expression of downstream signals affecting disease progression in SLE (82–85). Interestingly, many changes in methylation profiles of B cells observed in RA and SLE were also closely related to disease manifestations (86). In addition, alterations in the methylation profile of CD1C, TNFSF10, PARVG, NID1, DHRS12, ITPK1, ACSF3, and TNFRSF13C in RA patients can also predict the progression of RA (86). Furthermore, monocytes also have an important contribution to local and systemic inflammation and, many studies have shown a relation between changes in the monocyte methylation profile of RA and SLE patients with an increase in the level of pro-inflammatory factors (87). However, earlier studies mainly focused on the changes present only in cell methylation profile, hence, there are differences between the clinical markers we are proposing here from the previously reported ones. This is mainly due to our combined analysis of transcriptomics data with methylome profiles, which should be more reliable in predicting biomarker genes for diagnosis. Our data also show the prevailing inverse relationship between methylation status and transcription levels. If only the transcriptome data is used for analysis, the DEGs obtained will be related to the transcriptome only, which could be different from the results obtained by a combined analysis with methylome data. Therefore, we did this combined analysis by merging data to mainly increase the accuracy of the hub genes and its persuasiveness.

Both RA and SLE are polygenic and multifactorial in nature, therefore predictions based on a combined analysis of data might be more accurate and advantageous in predicting these diseases. Because of the paucity of the data, in this study, we only combined methylation profiles with transcriptomics data, hence we propose more such combined analyses of methylation pattern with other omics data may be required for predicting and treating diseases at personal level in the near future. At the same time, we would like to mention presence of few impediments in our investigation. Due to the lack of chip data, it is difficult to perform a complete comparative analysis of several autoimmune diseases at present. Therefore, we have selected RA and SLE, which are the most common, autoimmune rheumatic diseases, as the research objects for a combined analysis of genomic and epigenetic data sets. However, patients used in the gene-chip analysis were neither from homogenized disease sub-group nor belong to one geographic area. Furthermore, more immune cells like CD8^+^ T cells, neutrophils, NK cells, macrophages and other granulocytes were excluded from our study due to lack of open data sets available at present. Still, our study is expected to facilitate many such studies in the future involving different cells and data sets. Besides, it is important to mention that we validated abnormal methylome of gene expression by using DiseaseMeth 2.0 database only. Furthermore, we have collected patients’ samples from early phase of the disease before treatments were initiated, though it would be interesting to test samples from different phases of disease as well. In addition, due to small sample size, we did not observe any correlation between the proposed genes and clinical laboratory findings in the patients. So, to ascertain the usefulness of proposed genes as biomarkers more experiments are required using large cohort of patients at various phases of disease activity and treatment, and also to design drugs for targeting specific and distinct pathways operating in RA and SLE.

## Conclusions

Our study demonstrates unusually methylated DEGs and disease mechanisms of immune cells involved in RA and SLE using an extensive bioinformatics analysis by combining data from microarray analysis of gene expression and methylation, and found plausible molecular pathways involved in the development of RA and SLE at cellular level. We found 30 hub genes in different immune cell populations from RA and SLE data sets. We confirmed the expression of these hub genes using FACS sorted patients’ immune cells. Among them, 12 genes (BPTF, PHC2, JUN, KRAS, PTEN, FGFR2, ALB, SERB-1, SKP2, TUBA1A, IMP3, and SMAD4) of RA and 12 genes (OAS1, RSAD2, OASL, IFIT3, OAS2, IFIH1, CENPE, TOP2A, PBK, KIF11, IFIT1, and ISG15) of SLE were proposed as potential biomarkers based on ROC analysis for diagnosis. In addition, our analysis suggests that MAPK signaling pathway could potentially differentiate the mechanisms affecting T- and B- cells in RA, whereas, PI3K pathway may act as a common disease pathway affecting immune cells between RA and SLE. Compared to individual investigations and analysis, this study provides a more dependable and precise filtering of results by integrating appropriate data sets. However, more experiments are required to validate our inferences.

## Data Availability Statement

The raw data supporting the conclusions of this article will be made available by the authors, without undue reservation.

## Ethics Statement

The studies involving human participants were reviewed and approved by First Affiliated Hospital of Jinan University, Guangzhou, China. The patients/participants provided their written informed consent to participate in this study.

## Author Contributions

QF performed experiments, analyzed the data and prepared manuscript along with TL, PC, YW, TW, LM, and JO. KSN supervised the project and revised the manuscript. All authors contributed to the article and approved the submitted version.

## Funding

This project was supported by project grants from Southern Medical University, Guangzhou, China (Grant numbers C1034211, C1051004) given to KSN.

## Conflict of Interest

The authors declare that the research was conducted in the absence of any commercial or financial relationships that could be construed as a potential conflict of interest.
