# Supplementary material for: Comparative Analysis on Abnormal Methylome of Differentially Expressed Genes and Disease Pathways in the Immune Cells of RA and SLE
Source: Front Immunol. 2021 May 17;12:668007. doi: 10.3389/fimmu.2021.668007 (PMC8165287; doi:10.3389/fimmu.2021.668007)
Supplement: Supplementary file 1 [file DataSheet_1.docx]

**Table 1. Sample description and clinical laboratory findings**

|  | HC | | | | | | | | | | RA | | | | | | | | | | SLE | | | | | | | | | |
| --- | --- | --- | --- | --- | --- | --- | --- | --- | --- | --- | --- | --- | --- | --- | --- | --- | --- | --- | --- | --- | --- | --- | --- | --- | --- | --- | --- | --- | --- | --- |
| Patient number | 1 | 2 | 3 | 4 | 5 | 6 | 7 | 8 | 9 | 10 | 1 | 2 | 3 | 4 | 5 | 6 | 7 | 8 | 9 | 10 | 1 | 2 | 3 | 4 | 5 | 6 | 7 | 8 | 9 | 10 |
| Gender | F | F | F | M | M | F | M | F | F | M | F | M | F | M | F | F | M | M | F | M | F | F | F | F | M | M | F | M | M | F |
| Age (years) | 29 | 25 | 24 | 29 | 65 | 27 | 31 | 30 | 54 | 41 | 74 | 69 | 86 | 50 | 64 | 73 | 57 | 37 | 57 | 79 | 57 | 48 | 32 | 27 | 51 | 73 | 51 | 34 | 73 | 51 |
| Years of disease duration (years) | N/A | N/A | N/A | N/A | N/A | N/A | N/A | N/A | N/A | N/A | 13 | 20 | 0.5 | 2 | 9 | 2 | 4 | 3 | 6 | 9 | 16 | 2 | 2 | 4 | 7 | 6 | 9 | 1.5 | 6 | 10 |
| DAS28 | 0 | 0 | 0 | 0 | 0 | 0 | 0 | 0 | 0 | 0 | 4.78 | 4.59 | 5.45 | 1.83 | 4.13 | 5.64 | 6.80 | N/A | 3.91 | 6.08 | 0 | 0 | 0 | 0 | 0 | 0 | 0 | 0 | 0 | 0 |
| SLEDAI | 0 | 0 | 0 | 0 | 0 | 0 | 0 | 0 | 0 | 0 | 0 | 0 | 0 | 0 | 0 | 0 | 0 | 0 | 0 | 0 | 18 | 14 | 9 | 13 | 15 | 15 | 19 | 17 | 16 | 15 |
| Serum IgG (g/L) | N/A | N/A | N/A | N/A | N/A | N/A | N/A | N/A | N/A | N/A | 16.67 | 11.94 | 19.31 | 111.13 | 14.54 | 19.24 | 21.45 | 16.13 | 9.17 | 13.57 | 16.67 | 14.66 | 13.85 | 10.42 | 8.24 | 19.06 | 15.9 | 11.57 | 19.06 | 12.75 |
| Serum IgA (g/L) | N/A | N/A | N/A | N/A | N/A | N/A | N/A | N/A | N/A | N/A | 5.76 | 3.47 | 2.58 | 1.57 | 3.18 | 1.42 | 3.5 | 1.28 | 4.1 | 3.99 | 6.18 | 1.49 | 1.28 | 1.54 | 1.94 | 4.47 | 3.03 | 0.63 | 4.47 | 1.89 |
| Serum IgM (g/L) | N/A | N/A | N/A | N/A | N/A | N/A | N/A | N/A | N/A | N/A | 1.1 | 2 | 0.85 | 0.53 | 1.16 | 1.93 | 0.2 | 3.31 | 1.09 | 1.81 | 0.69 | 0.53 | 2.48 | 1.33 | 0.68 | 0.23 | 0.96 | 0.75 | 0.23 | 0.5 |
| C3 Complement (mg/L) | N/A | N/A | N/A | N/A | N/A | N/A | N/A | N/A | N/A | N/A | 1052.26 | 867.89 | 1386.04 | 1327 | 1272.46 | 1293 | 1870 | 1301 | 1214.83 | 1158.47 | 965.87 | 868 | 433 | 799.15 | 958.05 | 957 | 1009.31 | 968 | 957 | 934.09 |
| C4 Complement (mg/L) | N/A | N/A | N/A | N/A | N/A | N/A | N/A | N/A | N/A | N/A | 253.92 | 154.43 | 256.35 | 230 | 260.26 | 214 | 182 | 141 | 276.02 | 193.61 | 135.04 | 141 | 125 | 139.37 | 352.11 | 206 | 400.47 | 194 | 206 | 297.21 |
| C1q Complement (mg/L) | N/A | N/A | N/A | N/A | N/A | N/A | N/A | N/A | N/A | N/A | 180 | 197.8 | 221.3 | 189.8 | 187.7 | 222.5 | 183.2 | 17.6 | 175.4 | 207 | 233 | 151.3 | N/A | 212.3 | N/A | 100.6 | 191.3 | 170.3 | 100.6 | 144 |
| ASO (IU/ml) | N/A | N/A | N/A | N/A | N/A | N/A | N/A | N/A | N/A | N/A | 104.38 | 94.54 | 36.79 | 66.94 | 27.41 | 41.25 | 27.31 | 52.8 | 232.96 | 50.1 | 4.63 | 14.81 | N/A | 108.05 | N/A | 16.56 | 103.78 | 46.36 | 16.56 | 233.35 |
| RF (IU/ml) | N/A | N/A | N/A | N/A | N/A | N/A | N/A | N/A | N/A | N/A | 121.58 | 115.06 | 143.62 | 125.7 | 8.52 | 294.9 | 37.5 | 82.3 | 163.43 | 157.29 | 9.08 | 1.7 | N/A | 9.81 | N/A | 9.2 | 17.82 | 17.3 | 9.2 | 5.68 |
| ESR (mm/h) | N/A | N/A | N/A | N/A | N/A | N/A | N/A | N/A | N/A | N/A | 50 | 58 | 99 | 5 | 47 | 85 | 120 | N/A | 35 | 84 | 50 | 20 | 40 | 13 | 14 | 36 | 39 | 29 | 36 | 9 |
| CIC | N/A | N/A | N/A | N/A | N/A | N/A | N/A | N/A | N/A | N/A | 157 | 256 | 226 | 150 | 22 | 244 | 200 | 160 | 150 | 440 | 216 | 146 | 236 | 150 | 68 | 308 | 144 | 114 | N/A | 162 |
| CRP (mg/L) | N/A | N/A | N/A | N/A | N/A | N/A | N/A | N/A | N/A | N/A | 3 | 2.23 | 56.61 | 1.88 | 14.17 | 5.23 | 76.47 | 1.45 | 1.72 | 19.71 | 3.02 | 5.16 | 0.61 | 0.07 | 5.3 | 3.95 | 0.61 | 0.54 | 3.95 | 0.33 |
| Anti RA33 antibodies | － | － | － | － | － | － | － | － | － | － | － | N/A | － | － | － | － | － | － | － | － | － | － | － | － | N/A | － | － | － | N/A | N/A |
| Anti-CCP | － | － | － | － | － | － | － | － | － | － | ＋ | N/A | ＋ | ＋ | － | ＋ | ＋ | ＋ | ＋ | ＋ | ＋ | － | － | － | N/A | － | － | － | N/A | N/A |
| ANA | － | － | － | － | － | － | － | － | － | － | － | － | － | － | ＋ | － | － | ＋ | － | ＋ | ＋ | ＋ | ＋ | ＋ | ＋ | ＋ | ＋ | ＋ | ＋ | ＋ |
| Anti-ds-DNA | － | － | － | － | － | － | － | － | － | － | － | － | － | － | － | － | － | － | － | － | － | － | ＋ | － | － | － | － | － | － | － |
| ANUA | － | － | － | － | － | － | － | － | － | － | － | － | － | － | － | － | － | － | － | － | － | － | ＋ | － | － | － | － | － | － | － |
| Anti-SSA antibody | － | － | － | － | － | － | － | － | － | － | － | － | － | － | ＋ | － | － | － | － | － | ＋ | ＋ | － | ＋ | － | ＋ | ＋ | ＋ | ＋ | － |
| Anti-SSB antibody | － | － | － | － | － | － | － | － | － | － | － | － | － | － | － | － | － | － | － | － | ＋ | － | － | － | － | ＋ | － | ＋ | ＋ | － |
| Anti-Jo-1 antibodies | － | － | － | － | － | － | － | － | － | － | － | － | － | － | － | － | － | － | － | － | － | － | － | － | － | － | － | － | － | － |
| Anti-Scl 70 antibodies | － | － | － | － | － | － | － | － | － | － | － | － | － | － | － | － | － | － | － | － | － | － | － | － | － | － | － | － | － | － |
| Anti-UIRNP antibodies | － | － | － | － | － | － | － | － | － | － | － | － | － | － | － | － | － | － | － | － | ＋ | － | ＋ | － | － | － | － | － | － | ＋ |
| Anti-Ro52 Antibody | － | － | － | － | － | － | － | － | － | － | － | － | － | － | － | － | － | － | － | － | ＋ | ＋ | － | ＋ | － | ＋ | ＋ | ＋ | ＋ | － |
| Anti-Sm antibodies | － | － | － | － | － | － | － | － | － | － | － | － | － | － | － | － | － | － | － | － | － | － | ＋ | － | － | － | － | － | － | － |
| AMA-M2 | － | － | － | － | － | － | － | － | － | － | － | － | － | － | ＋ | － | － | － | － | － | － | － | － | － | － | － | － | － | － | － |
| Anti-PCNA antibody | － | － | － | － | － | － | － | － | － | － | － | － | － | － | － | － | － | － | N/A | － | － | － | － | － | － | － | － | － | － | － |

Note. F: Female, M: Male; SLE: Systemic lupus erythematosus; RA: Rheumatoid arthritis; CIC: Circulating immune complexes; CRP: C-reactive protein; ASO: Anti-streptolysin; ESR: Erythrocyte sedimentation rate; PCT: Procalcitonin; RF: Rheumatoid factor; Anti-CCP: Anti-cyclic citrullinated peptide; ANA: Anti-nuclear antibody; Anti-ds-DNA: Anti-dsDNA antibodies; ANUA: Anti-nucleosome antibody; AMA-M2: Anti-mitochondrial antibodies of M2 subtype; N/A: Not Applicable.

1. For anti-CCP antibody detection, anti-CCP antibody ELISA kit from German Oumeng Medical Diagnostic Company was used, and the cut off value > 5 IU/ml is considered as positive.
2. Anti RA33 antibodies, ANA, anti-ds-DNA, ANUA, anti-SSA antibody, anti-SSB antibody, anti-Jo-1 antibodies, anti-Scl 70 antibodies, anti-UIRNP antibodies, anti-Ro52 Antibody, anti-Sm antibodies, AMA-M2 and anti-PCNA antibody detection, ELISA Kits from German Oumeng Medical Diagnostic Company were used. Quantitative: < 20RU/ml is negative, ≥ 20RU/ml is positive.
3. DAS28: Disease Activity Score-28 for Rheumatoid Arthritis. Remission: DAS28 < 2.6; Low Disease Activity: 2.6 ≤ DAS28 ≤ 3.2; Moderate Disease Activity: 3.2 ＜ DAS28 ≤ 5.1; High Disease Activity: DAS28 > 5.1.
4. SLEDAI: SLE Disease Activity Index. No activity: 0 ≤ SLEDAI ≤ 4; Low Disease Activity: 5 ≤ SLEDAI ≤ 9; Moderate Disease Activity: 10 ≤ SLEDAI ≤ 14; High Disease Activity: SLEDAI ≥15.

**Table 2. RT-qPCR primers used in this study**

| RA | | | SLE | | | |
| --- | --- | --- | --- | --- | --- | --- |
| CD19^+^ B cells | | | | | | |
| Gene name |  | Sequence 5’-3’ | | Gene name |  | Sequence 5’-3’ |
| BPTF | F | TTCCAGTTAAGCATCAGGTT | | OAS1 | F | GAGAGTTCATCCAGGAAATTAGG |
|  | R | GGTCCTTCTTCTCAGTAATCT | |  | R | GAAGTCTCTCTGTAGTTCTGTGA |
| MRPS28 | F | ATCCGAAAGTGGTAGTTCCAAT | | XAF1 | F | ATGTAGTCTCTGCCAACTTCAC |
|  | R | GCATTAGCCTCTAGTACAGTTG | |  | R | CAGTAGATTTCCCTTTCAGGAG |
| SF1 | F | CCAGGAATGCCTACAGTTAT | | RSAD2 | F | TAGGGATTATAGAGTCGCTTTCAAG |
|  | R | ATCTCTGTGATGAGGTTGTG | |  | R | TAGGAGTCTTTCATCTTCTGGTTAG |
| PSAP | F | ATTGCTATCCAGATGATGATGC | | MX1 | F | CTATTACTGAATGGAGATGCTACTG |
|  | R | CTCAGTCTTGTTGTTGTCAATC | |  | R | GGTTATGCCAGGAAGGTCTATTAG |
| HNRNPC | F | GACTATGACTTTCAACGGGACTAT | | STAT1 | F | AATCTGTCCTTCTTCCTGACTC |
|  | R | CCTCAGACTCCATCTTCACATTAG | |  | R | GTTCTTTCTTCGTGTAGGGTTC |
| ICT1 | F | TGAGTTCAAGAGCATCTACAG | | OASL | F | ACTGATGCAGGAACTGTATAG |
|  | R | TATGCGTGATGGCTATCTTC | |  | R | ATCAGCCTCAGAACATCTTTG |
| PHC2 | F | GATTTCTGTGTACAGTGGTATTCC | | IFIT3 | F | GGAACTTATTCAAGGAAGACAG |
|  | R | GTGCTTCCTTGTCTATTGGATAC | |  | R | CATAAATCTGAGCATCTGAGAG |
| JARID2 | F | CACCTTCCATACATTGACTACT | | OAS3 | F | GCTGTGATTCTGAACTTGTCATC |
|  | R | GCAATCTGGTAGAGTAACTTCT | |  | R | CTAACATCCATCCAGTCCTCAAG |
| DICER1 | F | GGTTACCATATCCATTGAGTTG | | OAS2 | F | CTTCTGGATAAGTTCATCAAGGAG |
|  | R | TAAGAGGTAGAACACAGTATGC | |  | R | TTGAGGACTTTGGATTTCAGAGAG |
| ERBB2 | F | GTCCCTACAACTACCTTTCTAC | | IFIH1 | F | GGAGCAATATACTAGGACTGAG |
|  | R | ATGCTGAGATGTATAGGTAACC | |  | R | GAGACCATAACGGATAACAATG |
| CD4^+^ T cells | | | | | | |
| STAT5B | F | ACCATCACGGACATTATCTC | | CENPE | F | CTTCGTGCTGACTATGATAATCTG |
|  | R | ATCTCGCCACTGTAATCATT | |  | R | CTGTAGCTTCTTAATCTGGTCTTC |
| SOCS3 | F | GGAATGCTGAACTAATGAGAAC | | MELK | F | CTCCTAAGTCTGCTGTAAAGAATG |
|  | R | CAGGTACAGGAAGGTATAGTATG | |  | R | CTGTCATTAACTTGTCTGTTCCTG |
| JUN | F | GCGGACCTTATGGCTACAGTAACC | | NCAPG | F | CTTTAGAACTCAGTAGCCATCTTG |
|  | R | CTGGGTTGAAGTTGCTGAGGTTTG | |  | R | TCTCTGTCCTCTGTTAGTCTTTAG |
| STAT1 | F | AATCTGTCCTTCTTCCTGACTC | | AURKA | F | GCTACCAGAGTCTACCTAATTC |
|  | R | GTTCTTTCTTCGTGTAGGGTTC | |  | R | GGATTATGCTTCAACAGTCTTG |
| KRAS | F | AACCTGTCTCTTGGATATTCTC | | BUB1 | F | GTCTATGTCCATCACCTTCTTG |
|  | R | GTATAGAAGGCATCATCAACAC | |  | R | TCCTACTAATACACTGCCATTC |
| PTEN | F | CGAACTGGTGTAATGATATGTG | | TOP2A | F | ACGGTGTTGGATATTCTAAGAG |
|  | R | GGTCCTGGTATGAAGAATGTAT | |  | R | AGAAGATAGTTGAAGGTTGGTC |
| FGFR2 | F | GATGGAGATGATGAAGATGATTGG | | CCNB1 | F | TCTAAGATTGGAGAGGTTGATG |
|  | R | GAACACGGTTAATGTCATAGGAG | |  | R | GCATAACTGGAAGAAGAGATTC |
| DICER1 | F | GGTTACCATATCCATTGAGTTG | | KIF23 | F | GATGTCCTAAGAGAGAACCAAATG |
|  | R | TAAGAGGTAGAACACAGTATGC | |  | R | TACAGGTCTTGCTACTTCAACTTC |
| ALB | F | CAAGAACCTGAGAGAAATGAATGC | | PBK | F | GAGGAGAATGGTGTTATTACTG |
|  | R | CTGTCACTAACTTGGAAACTTCTG | |  | R | CATTAGTGCATACAGAGAAGAG |
| CD44 | F | CAAGGATGACTGATGTAGACAGAAATG | | KIF11 | F | GAAACTACGATTGATGGAGAAGAG |
|  | R | CATTCCTATTGCTTGATGTCAGAGTAG | |  | R | GAGCATATTCCAATGTACTCAGAG |
| CD14^+^ monocytes | | | | | | |
| RPL15 | F | CTATGGAGAAAGAAGCAGTCTG | | IFIT1 | F | GGAGCAATATACTAGGACTGAG |
|  | R | GAATGGATCAATGAGGATAACC | |  | R | GAGACCATAACGGATAACAATG |
| SOCS3 | F | GGAATGCTGAACTAATGAGAAC | | ISG15 | F | ATGTCGGTGTCAGAGCTGAA |
|  | R | CAGGTACAGGAAGGTATAGTATG | |  | R | AGGATGCTCAGAGGTTCGTC |
| SIRT1 | F | CCATCTCTCTGTCACAAATTCATAG | | MX1 | F | CTTGTGAACGAAGATAAGTGGAG |
|  | R | CAATAACAATGAGGAGGTCAACTTC | |  | R | GTTATGCCAGGAAGGTCTATTAG |
| SERBP1 | F | GAAAGAAGGAATAAGACGAGTTGG | | OAS2 | F | CTTCTGGATAAGTTCATCAAGGAG |
|  | R | TCTATCACTTCCACTATGCCTATC | |  | R | TTGAGGACTTTGGATTTCAGAGAG |
| SKP2 | F | CTAAAGGTCTCTGGTGTTTGTAAG | | OASL | F | ACTGATGCAGGAACTGTATAG |
|  | R | GTGGACACTTCTATAACTGAGTTC | |  | R | ATCAGCCTCAGAACATCTTTG |
| TUBA1A | F | CATGGAACGTCTCTCAGTTGATTATG | | IFIT2 | F | GAAGGAGAGAAGTTAGTTGAAG |
|  | R | TTAGTATTCCTCTCCTTCTTCCTCAC | |  | R | GACACGGAAGAGATTATCATTG |
| IMP3 | F | TTAAGTTCCACGAGCAGAAG | | IFIT3 | F | GGAACTTATTCAAGGAAGACAG |
|  | R | CGCTCCTCATTGTACTCTAG | |  | R | CATAAATCTGAGCATCTGAGAG |
| EXOSC5 | F | GGTGAAGGTCAGCAAAGAGATT | | OAS1 | F | GAGAGTTCATCCAGGAAATTAGG |
|  | R | GGTAGAAACGGAAGACGTGTTG | |  | R | GAAGTCTCTCTGTAGTTCTGTGA |
| SMAD4 | F | CTACTTACCATCATAACAGCACTAC | | RSAD2 | F | GTATTTGGACATTCTCGCTATCTC |
|  | R | CTTAAATGTCTCTCCTACCTGAAC | |  | R | AGGAGTCTTTCATCTTCTGGTTAG |
| KRAS | F | AACCTGTCTCTTGGATATTCTC | | OAS3 | F | GCTGTGATTCTGAACTTGTCATC |
|  | R | GTATAGAAGGCATCATCAACAC | |  | R | CTAACATCCATCCAGTCCTCAAG |

**Table 3. Gene ontology analysis of abnormally methylated-DEGs in CD19^+^ B-, CD4^+^ T- cells and CD14^+^ monocytes**

| Cell type | Diseases | Category | Term | Count | % | P value |
| --- | --- | --- | --- | --- | --- | --- |
| CD19^+^ B cells | RA | BP | GO:0006351~transcription, DNA-templated | 35 | 20.83333333 | 1.62E-04 |
|  |  | BP | GO:0001889~liver development | 5 | 2.976190476 | 4.76E-03 |
|  |  | BP | GO:0006355~regulation of transcription, DNA-templated | 25 | 14.88095238 | 5.22E-03 |
|  |  | BP | GO:0006309~apoptotic DNA fragmentation | 3 | 1.785714286 | 8.11E-03 |
|  |  | BP | GO:0048844~artery morphogenesis | 3 | 1.785714286 | 0.015653998 |
|  |  | CC | GO:0005634~nucleus | 77 | 45.83333333 | 5.91E-07 |
|  |  | CC | GO:0005654~nucleoplasm | 49 | 29.16666667 | 9.24E-07 |
|  |  | CC | GO:0005737~cytoplasm | 62 | 36.9047619 | 3.65E-03 |
|  |  | CC | GO:0005730~nucleolus | 16 | 9.523809524 | 0.007214508 |
|  |  | CC | GO:0016363~nuclear matrix | 5 | 2.976190476 | 0.009886249 |
|  |  | MF | GO:0005515~protein binding | 109 | 64.88095238 | 5.46E-06 |
|  |  | MF | GO:0044822~poly(A) RNA binding | 24 | 14.28571429 | 2.57E-04 |
|  |  | MF | GO:0003700~transcription factor activity, sequence-specific DNA binding | 21 | 12.5 | 5.13E-04 |
|  |  | MF | GO:0003677~DNA binding | 30 | 17.85714286 | 5.89E-04 |
|  |  | MF | GO:0003730~mRNA 3'-UTR binding | 5 | 2.976190476 | 1.05E-03 |
|  | SLE | BP | GO:0060337~type I interferon signaling pathway | 18 | 10.11235955 | 3.57E-20 |
|  |  | BP | GO:0051607~defense response to virus | 20 | 11.23595506 | 3.47E-15 |
|  |  | BP | GO:0009615~response to virus | 16 | 8.988764045 | 2.53E-13 |
|  |  | BP | GO:0045071~negative regulation of viral genome replication | 11 | 6.179775281 | 4.73E-12 |
|  |  | BP | GO:0060333~interferon-gamma-mediated signaling pathway | 9 | 5.056179775 | 5.05E-07 |
|  |  | CC | GO:0005829~cytosol | 61 | 34.26966292 | 2.48E-07 |
|  |  | CC | GO:0048471~perinuclear region of cytoplasm | 18 | 10.11235955 | 1.04E-04 |
|  |  | CC | GO:0005737~cytoplasm | 71 | 39.88764045 | 7.86E-04 |
|  |  | CC | GO:0016020~membrane | 35 | 19.66292135 | 0.003289273 |
|  |  | CC | GO:0005764~lysosome | 8 | 4.494382022 | 0.006261331 |
|  |  | MF | GO:0003725~double-stranded RNA binding | 8 | 4.494382022 | 2.28E-06 |
|  |  | MF | GO:0001730~2'-5'-oligoadenylate synthetase activity | 4 | 2.247191011 | 3.78E-06 |
|  |  | MF | GO:0005515~protein binding | 103 | 57.86516854 | 0.011287437 |
|  |  | MF | GO:0042803~protein homodimerization activity | 15 | 8.426966292 | 0.013725703 |
|  |  | MF | GO:0005525~GTP binding | 10 | 5.617977528 | 0.014337113 |
| CD4^+^ T cells | RA | BP | GO:0001525~angiogenesis | 9 | 5.056179775 | 0.001524023 |
|  |  | BP | GO:0008360~regulation of cell shape | 7 | 3.93258427 | 0.002456981 |
|  |  | BP | GO:0000122~negative regulation of transcription from RNA polymerase II promoter | 16 | 8.988764045 | 0.00442377 |
|  |  | BP | GO:0030336~negative regulation of cell migration | 5 | 2.808988764 | 0.013759877 |
|  |  | BP | GO:0071320~cellular response to cAMP | 4 | 2.247191011 | 0.014017888 |
|  |  | CC | GO:0005737~cytoplasm | 80 | 44.94382022 | 5.72E-07 |
|  |  | CC | GO:0005634~nucleus | 76 | 42.69662921 | 4.54E-05 |
|  |  | CC | GO:0005654~nucleoplasm | 45 | 25.28089888 | 2.24E-04 |
|  |  | CC | GO:0030424~axon | 9 | 5.056179775 | 0.001158043 |
|  |  | CC | GO:0005794~Golgi apparatus | 17 | 9.550561798 | 0.006929818 |
|  |  | MF | GO:0005515~protein binding | 120 | 67.41573034 | 5.48E-08 |
|  |  | MF | GO:0044822~poly(A) RNA binding | 24 | 13.48314607 | 5.86E-04 |
|  |  | MF | GO:0046966~thyroid hormone receptor binding | 4 | 2.247191011 | 0.002218692 |
|  |  | MF | GO:0005524~ATP binding | 27 | 15.16853933 | 0.002536562 |
|  |  | MF | GO:0000166~nucleotide binding | 10 | 5.617977528 | 0.007036487 |
|  | SLE | BP | GO:0007059~chromosome segregation | 9 | 2.195121951 | 1.47E-04 |
|  |  | BP | GO:0000278~mitotic cell cycle | 7 | 1.707317073 | 3.01E-04 |
|  |  | BP | GO:0006915~apoptotic process | 26 | 6.341463415 | 0.00124767 |
|  |  | BP | GO:0050796~regulation of insulin secretion | 7 | 1.707317073 | 0.004083754 |
|  |  | BP | GO:0035556~intracellular signal transduction | 19 | 4.634146341 | 0.005077641 |
|  |  | CC | GO:0005737~cytoplasm | 173 | 42.19512195 | 1.33E-10 |
|  |  | CC | GO:0005829~cytosol | 112 | 27.31707317 | 6.86E-07 |
|  |  | CC | GO:0016020~membrane | 69 | 16.82926829 | 0.001545665 |
|  |  | CC | GO:0005819~spindle | 9 | 2.195121951 | 0.004871346 |
|  |  | CC | GO:0005654~nucleoplasm | 80 | 19.51219512 | 0.006423487 |
|  |  | MF | GO:0005515~protein binding | 239 | 58.29268293 | 7.28E-06 |
|  |  | MF | GO:0019901~protein kinase binding | 20 | 4.87804878 | 8.36E-04 |
|  |  | MF | GO:0005524~ATP binding | 52 | 12.68292683 | 0.001525391 |
|  |  | MF | GO:0019899~enzyme binding | 16 | 3.902439024 | 0.008132629 |
|  |  | MF | GO:0046872~metal ion binding | 63 | 15.36585366 | 0.009438166 |
| CD14^+^ monocytes | RA | BP | GO:0016032~viral process | 11 | 5.699481865 | 0.001736387 |
|  |  | BP | GO:0008283~cell proliferation | 12 | 6.21761658 | 2.39E-03 |
|  |  | BP | GO:0045944~positive regulation of transcription from RNA polymerase II promoter | 22 | 11.39896373 | 0.002537249 |
|  |  | BP | GO:0043433~negative regulation of sequence-specific DNA binding transcription factor activity | 5 | 2.590673575 | 0.004219181 |
|  |  | BP | GO:0006112~energy reserve metabolic process | 3 | 1.554404145 | 0.011416845 |
|  |  | CC | GO:0005739~mitochondrion | 33 | 17.0984456 | 5.90E-06 |
|  |  | CC | GO:0005737~cytoplasm | 78 | 40.41450777 | 1.87E-04 |
|  |  | CC | GO:0016605~PML body | 7 | 3.626943005 | 5.34E-04 |
|  |  | CC | GO:0005730~nucleolus | 21 | 10.88082902 | 5.65E-04 |
|  |  | CC | GO:0032993~protein-DNA complex | 4 | 2.07253886 | 0.002102381 |
|  |  | MF | GO:0005515~protein binding | 124 | 64.24870466 | 6.40E-05 |
|  |  | MF | GO:0008134~transcription factor binding | 12 | 6.21761658 | 3.20E-04 |
|  |  | MF | GO:0044822~poly(A) RNA binding | 24 | 12.43523316 | 0.003191233 |
|  |  | MF | GO:0070491~repressing transcription factor binding | 4 | 2.07253886 | 0.006648475 |
|  |  | MF | GO:0000978~RNA polymerase II core promoter proximal region sequence-specific DNA binding | 10 | 5.18134715 | 0.017157239 |
|  | SLE | BP | GO:0060337~type I interferon signaling pathway | 27 | 6.923076923 | 1.17E-26 |
|  |  | BP | GO:0051607~defense response to virus | 33 | 8.461538462 | 2.88E-21 |
|  |  | BP | GO:0045071~negative regulation of viral genome replication | 19 | 4.871794872 | 8.16E-20 |
|  |  | BP | GO:0060333~interferon-gamma-mediated signaling pathway | 21 | 5.384615385 | 3.92E-17 |
|  |  | BP | GO:0009615~response to virus | 24 | 6.153846154 | 2.20E-16 |
|  |  | CC | GO:0005829~cytosol | 131 | 33.58974359 | 7.21E-14 |
|  |  | CC | GO:0005737~cytoplasm | 167 | 42.82051282 | 4.26E-10 |
|  |  | CC | GO:0005654~nucleoplasm | 102 | 26.15384615 | 1.08E-08 |
|  |  | CC | GO:0016020~membrane | 76 | 19.48717949 | 1.39E-05 |
|  |  | CC | GO:0048471~perinuclear region of cytoplasm | 30 | 7.692307692 | 5.51E-05 |
|  |  | MF | GO:0005515~protein binding | 253 | 64.87179487 | 1.89E-09 |
|  |  | MF | GO:0001730~2'-5'-oligoadenylate synthetase activity | 4 | 1.025641026 | 4.31E-05 |
|  |  | MF | GO:0044822~poly(A) RNA binding | 47 | 12.05128205 | 5.23E-05 |
|  |  | MF | GO:0032403~protein complex binding | 16 | 4.102564103 | 6.11E-05 |
|  |  | MF | GO:0003725~double-stranded RNA binding | 8 | 2.051282051 | 3.99E-04 |

Note: BP, Biological Process; CC, Cellular Compartment; MF, Molecular Function.

**Table 4. KEGG pathway analysis of aberrantly methylated-DEGs in CD19^+^ B-, CD4^+^ T cells and** **CD14^+^ monocytes**

| Cell type | Diseases | Pathway ID | Pathway name | Gen no. | % | P value | Genes |
| --- | --- | --- | --- | --- | --- | --- | --- |
| CD19^+^ B cells | RA | hsa04010 | MAPK signaling pathway | 8 | 4.761904762 | 0.009455073 | HSPA2, RAC2, ELK4, JUND, MKNK2, PPM1B, RASA1, NFATC1 |
|  | SLE | hsa05162 | Measles | 11 | 6.179775281 | 6.52E-06 | IFIH1, CCND2, OAS1, STAT1, OAS2, OAS3, MX1, IRF7, FAS, EIF2AK2, SLAMF1 |
|  |  | hsa05164 | Influenza A | 11 | 6.179775281 | 6.79E-05 | IFIH1, MAP2K2, RSAD2, OAS1, STAT1, OAS2, OAS3, MX1, IRF7, FAS, EIF2AK2 |
|  |  | hsa05168 | Herpes simplex infection | 10 | 5.617977528 | 5.12E-04 | IFIH1, SP100, OAS1, STAT1, OAS2, OAS3, IRF7, FAS, TAP1, EIF2AK2 |
|  |  | hsa05219 | Bladder cancer | 5 | 2.808988764 | 0.001764838 | MAP2K2, MMP2, E2F3, TYMP, HBEGF |
|  |  | hsa05160 | Hepatitis C | 6 | 3.370786517 | 0.027367677 | OAS1, STAT1, OAS2, OAS3, IRF7, EIF2AK2 |
|  |  | hsa05200 | Pathways in cancer | 11 | 6.179775281 | 0.027737112 | LAMA5, ARHGEF12, MAP2K2, CTBP2, STAT1, MMP2, LAMB4, FAS, E2F3, LAMC1, BRCA2 |
|  |  | hsa04915 | Estrogen signaling pathway | 5 | 2.808988764 | 0.037571897 | GABBR1, MAP2K2, MMP2, ESR2, HBEGF |
|  |  | hsa05161 | Hepatitis B | 6 | 3.370786517 | 0.037708456 | IFIH1, MAP2K2, STAT1, IRF7, FAS, E2F3 |
|  |  | hsa05145 | Toxoplasmosis | 5 | 2.808988764 | 0.051915951 | LAMA5, STAT1, ALOX5, LAMB4, LAMC1 |
|  |  | hsa04917 | Prolactin signaling pathway | 4 | 2.247191011 | 0.062104849 | MAP2K2, CCND2, STAT1, ESR2 |
|  |  | hsa05222 | Small cell lung cancer | 4 | 2.247191011 | 0.094726892 | LAMA5, LAMB4, E2F3, LAMC1 |
|  |  | hsa04640 | Hematopoietic cell lineage | 4 | 2.247191011 | 0.099868564 | FCER2, CR1, IL4R, CD1C |
| CD4^+^ T cells | RA | hsa05205 | Proteoglycans in cancer | 9 | 5.056179775 | 0.001076735 | PIK3CG, EZR, KRAS, CD44, PPP1R12A, WNT11, PRKACB, TGFB2, ITPR2 |
|  |  | hsa04919 | Thyroid hormone signaling pathway | 7 | 3.93258427 | 0.001219553 | PIK3CG, KRAS, NCOA2, MED30, MED16, PRKACB, STAT1 |
|  |  | hsa04915 | Estrogen signaling pathway | 6 | 3.370786517 | 0.003643443 | HSPA1L, PIK3CG, KRAS, JUN, PRKACB, ITPR2 |
|  |  | hsa05161 | Hepatitis B | 7 | 3.93258427 | 0.003940313 | PIK3CG, KRAS, JUN, STAT5B, STAT1, PTEN, TGFB2 |
|  |  | hsa04917 | Prolactin signaling pathway | 5 | 2.808988764 | 0.00632275 | PIK3CG, KRAS, SOCS3, STAT5B, STAT1 |
|  |  | hsa05166 | HTLV-I infection | 8 | 4.494382022 | 0.016441357 | PIK3CG, KRAS, JUN, STAT5B, WNT11, PRKACB, MYB, TGFB2 |
|  |  | hsa05200 | Pathways in cancer | 10 | 5.617977528 | 0.020639396 | PIK3CG, FGFR2, KRAS, JUN, STAT5B, WNT11, PRKACB, STAT1, PTEN, TGFB2 |
|  |  | hsa05210 | Colorectal cancer | 4 | 2.247191011 | 0.026777121 | PIK3CG, KRAS, JUN, TGFB2 |
|  |  | hsa05230 | Central carbon metabolism in cancer | 4 | 2.247191011 | 0.029067458 | PIK3CG, FGFR2, KRAS, PTEN |
|  |  | hsa05212 | Pancreatic cancer | 4 | 2.247191011 | 0.030250521 | PIK3CG, KRAS, STAT1, TGFB2 |
|  |  | hsa05211 | Renal cell carcinoma | 4 | 2.247191011 | 0.03145878 | PIK3CG, KRAS, JUN, TGFB2 |
|  |  | hsa05220 | Chronic myeloid leukemia | 4 | 2.247191011 | 0.039233703 | PIK3CG, KRAS, STAT5B, TGFB2 |
|  |  | hsa04380 | Osteoclast differentiation | 5 | 2.808988764 | 0.047886616 | PIK3CG, SOCS3, JUN, STAT1, TGFB2 |
|  |  | hsa04010 | MAPK signaling pathway | 7 | 3.93258427 | 0.048347412 | HSPA1L, FGFR2, KRAS, PTPN5, JUN, PRKACB, TGFB2 |
|  |  | hsa04068 | FoxO signaling pathway | 5 | 2.808988764 | 0.051286071 | PIK3CG, KRAS, FBXO32, PTEN, TGFB2 |
|  |  | hsa04024 | cAMP signaling pathway | 6 | 3.370786517 | 0.05572455 | PIK3CG, ATP2B2, ACOX1, JUN, PPP1R12A, PRKACB |
|  |  | hsa04012 | ErbB signaling pathway | 4 | 2.247191011 | 0.062490738 | PIK3CG, KRAS, JUN, STAT5B |
|  |  | hsa05203 | Viral carcinogenesis | 6 | 3.370786517 | 0.06284399 | PIK3CG, KRAS, JUN, STAT5B, PRKACB, HDAC9 |
|  |  | hsa05215 | Prostate cancer | 4 | 2.247191011 | 0.064227241 | PIK3CG, FGFR2, KRAS, PTEN |
|  |  | hsa04810 | Regulation of actin cytoskeleton | 6 | 3.370786517 | 0.068232943 | PIK3CG, FGFR2, EZR, KRAS, ARHGEF7, PPP1R12A |
|  |  | hsa04912 | GnRH signaling pathway | 4 | 2.247191011 | 0.06957 | KRAS, JUN, PRKACB, ITPR2 |
|  |  | hsa04921 | Oxytocin signaling pathway | 5 | 2.808988764 | 0.071594404 | KRAS, JUN, PPP1R12A, PRKACB, ITPR2 |
|  |  | hsa04022 | cGMP-PKG signaling pathway | 5 | 2.808988764 | 0.083091641 | MEF2D, ATP2B2, SLC8A1, PPP1R12A, ITPR2 |
|  |  | hsa04014 | Ras signaling pathway | 6 | 3.370786517 | 0.087160222 | PIK3CG, FGFR2, KRAS, PLA2G12A, PRKACB, RASA3 |
|  |  | hsa05146 | Amoebiasis | 4 | 2.247191011 | 0.099115642 | PIK3CG, C9, PRKACB, TGFB2 |
|  | SLE | hsa05340 | Primary immunodeficiency | 5 | 1.219512195 | 0.014934376 | CD79A, DCLRE1C, RFX5, BLNK, UNG |
|  |  | hsa00670 | One carbon pool by folate | 4 | 0.975609756 | 0.017920702 | MTFMT, MTHFD1, MTHFD2, TYMS |
|  |  | hsa00240 | Pyrimidine metabolism | 8 | 1.951219512 | 0.024160891 | RRM2, PNPT1, NME7, PRIM1, POLR1B, TYMS, DCTPP1, NME1 |
|  |  | hsa03430 | Mismatch repair | 4 | 0.975609756 | 0.026168743 | RFC5, MSH3, RFC2, MLH3 |
|  |  | hsa04510 | Focal adhesion | 12 | 2.926829268 | 0.031926579 | VAV3, PAK1, SHC3, COL5A1, LAMA3, COL11A2, PTEN, FN1, COL6A3, TLN2, PIK3R1, PAK4 |
|  |  | hsa00970 | Aminoacyl-tRNA biosynthesis | 6 | 1.463414634 | 0.038571875 | CARS, YARS, DARS, MTFMT, IARS, AARS |
|  |  | hsa04662 | B cell receptor signaling pathway | 6 | 1.463414634 | 0.045362998 | VAV3, CD79B, CD79A, BLNK, DAPP1, PIK3R1 |
|  |  | hsa04930 | Type II diabetes mellitus | 5 | 1.219512195 | 0.046236606 | SOCS2, CACNA1B, CACNA1D, CACNA1C, PIK3R1 |
|  |  | hsa05134 | Legionellosis | 5 | 1.219512195 | 0.066064014 | CASP7, CASP1, SEC22B, TLR5, HSPD1 |
|  |  | hsa03030 | DNA replication | 4 | 0.975609756 | 0.080944065 | RFC5, RNASEH2B, RFC2, PRIM1 |
|  |  | hsa04210 | Apoptosis | 5 | 1.219512195 | 0.09827607 | CASP7, CASP10, TNFSF10, PIK3R1, TNFRSF10D |
| CD14^+^ monocytes | RA | hsa05210 | Colorectal cancer | 5 | 2.590673575 | 0.011498646 | MSH6, KRAS, SMAD4, LEF1, TCF7L2 |
|  |  | hsa05130 | Pathogenic Escherichia coli infection | 4 | 2.07253886 | 0.035684041 | TUBB2A, NCK1, ARPC5L, TUBA1A |
|  |  | hsa03060 | Protein export | 3 | 1.554404145 | 0.041858659 | OXA1L, SRPRB, SRP9 |
|  |  | hsa05221 | Acute myeloid leukemia | 4 | 2.07253886 | 0.045162887 | KRAS, PIM1, LEF1, TCF7L2 |
|  |  | hsa05216 | Thyroid cancer | 3 | 1.554404145 | 0.063600078 | KRAS, LEF1, TCF7L2 |
|  |  | hsa04917 | Prolactin signaling pathway | 4 | 2.07253886 | 0.080133581 | KRAS, SOCS3, IRF1, SOCS5 |
|  |  | hsa04142 | Lysosome | 5 | 2.590673575 | 0.093637447 | LAPTM4B, SLC11A1, SLC17A5, PLA2G15, ENTPD4 |
|  |  | hsa04612 | Antigen processing and presentation | 4 | 2.07253886 | 0.093785268 | NFYB, PSME3, HLA-DMB, HLA-DRA |
|  | SLE | hsa05164 | Influenza A | 24 | 6.153846154 | 1.87E-10 | CIITA, RSAD2, DDX58, PRKCB, STAT1, STAT2, MX1, EIF2AK2, ADAR, PRKCA, MAPK14, PIK3CG, PML, HLA-DMA, HLA-DMB, OAS1, OAS2, OAS3, IRF7, FAS, JAK2, MAP2K7, MAP2K6, HLA-DPA1 |
|  |  | hsa05168 | Herpes simplex infection | 23 | 5.897435897 | 2.99E-09 | SP100, DDX58, STAT1, STAT2, HLA-B, CUL1, TAP2, EIF2AK2, TAP1, HLA-A, IFIT1, HCFC2, PML, HLA-E, HLA-DMA, HLA-DMB, OAS1, OAS2, OAS3, IRF7, FAS, JAK2, HLA-DPA1 |
|  |  | hsa05169 | Epstein-Barr virus infection | 17 | 4.358974359 | 1.44E-07 | PSMD14, DDX58, STAT3, HLA-B, EIF2AK2, HLA-A, MAPK14, PIK3CG, HLA-E, NCOR2, MYC, PSMC2, MAP2K7, MAP3K14, CD44, MAP2K6, HLA-DPA1 |
|  |  | hsa05162 | Measles | 14 | 3.58974359 | 5.67E-05 | DDX58, STAT1, STAT2, STAT3, MX1, EIF2AK2, ADAR, PIK3CG, OAS1, OAS2, OAS3, IRF7, FAS, JAK2 |
|  |  | hsa05332 | Graft-versus-host disease | 7 | 1.794871795 | 2.17E-04 | HLA-DMA, HLA-DMB, HLA-B, FAS, HLA-A, HLA-DPA1, HLA-E |
|  |  | hsa05160 | Hepatitis C | 13 | 3.333333333 | 2.36E-04 | DDX58, STAT1, STAT2, STAT3, EIF2AK2, PPP2R2A, IFIT1, MAPK14, PIK3CG, OAS1, OAS2, OAS3, IRF7 |
|  |  | hsa05330 | Allograft rejection | 7 | 1.794871795 | 4.17E-04 | HLA-DMA, HLA-DMB, HLA-B, FAS, HLA-A, HLA-DPA1, HLA-E |
|  |  | hsa04940 | Type I diabetes mellitus | 7 | 1.794871795 | 8.41E-04 | HLA-DMA, HLA-DMB, HLA-B, FAS, HLA-A, HLA-DPA1, HLA-E |
|  |  | hsa04612 | Antigen processing and presentation | 9 | 2.307692308 | 9.62E-04 | HLA-DMA, CIITA, HLA-DMB, HLA-B, TAP2, TAP1, HLA-A, HLA-DPA1, HLA-E |
|  |  | hsa05320 | Autoimmune thyroid disease | 7 | 1.794871795 | 0.002610208 | HLA-DMA, HLA-DMB, HLA-B, FAS, HLA-A, HLA-DPA1, HLA-E |
|  |  | hsa04380 | Osteoclast differentiation | 11 | 2.820512821 | 0.002785369 | CYLD, STAT1, STAT2, CTSK, BLNK, PPARG, MAPK14, MAP2K7, MAP3K14, PIK3CG, MAP2K6 |
|  |  | hsa05205 | Proteoglycans in cancer | 14 | 3.58974359 | 0.002890488 | FZD5, PRKCB, SDC2, STAT3, PRKCA, MAPK14, PIK3CG, RRAS, MYC, FAS, HPSE, EZR, MET, CD44 |
|  |  | hsa05161 | Hepatitis B | 11 | 2.820512821 | 0.005753946 | DDX58, STAT1, YWHAB, PRKCB, MYC, STAT2, STAT3, IRF7, FAS, PRKCA, PIK3CG |
|  |  | hsa04010 | MAPK signaling pathway | 15 | 3.846153846 | 0.008418505 | PRKCB, DUSP1, CACNA2D3, PLA2G4A, PRKCA, ARRB1, MAPK14, PPM1B, RRAS, MYC, FAS, MAP2K7, MAP3K14, FGFR2, MAP2K6 |
|  |  | hsa05166 | HTLV-I infection | 15 | 3.846153846 | 0.008702564 | RANBP1, IL15RA, FZD5, HLA-B, HLA-A, PIK3CG, HLA-E, HLA-DMA, HLA-DMB, APC, RRAS, MYC, DVL3, MAP3K14, HLA-DPA1 |
|  |  | hsa05145 | Toxoplasmosis | 9 | 2.307692308 | 0.009617207 | HLA-DMA, CIITA, HLA-DMB, STAT1, STAT3, JAK2, MAPK14, MAP2K6, HLA-DPA1 |
|  |  | hsa04664 | Fc epsilon RI signaling pathway | 7 | 1.794871795 | 0.009839285 | PRKCB, PLA2G4A, PRKCA, MAPK14, MAP2K7, PIK3CG, MAP2K6 |
|  |  | hsa05416 | Viral myocarditis | 6 | 1.538461538 | 0.01842056 | HLA-DMA, HLA-DMB, HLA-B, HLA-A, HLA-DPA1, HLA-E |
|  |  | hsa05200 | Pathways in cancer | 19 | 4.871794872 | 0.01924107 | TCF7L2, FZD5, PRKCB, DAPK1, STAT1, GSTP1, STAT3, PRKCA, FOXO1, PIK3CG, PML, RBX1, APC, MYC, DVL3, FAS, PPARG, MET, FGFR2 |
|  |  | hsa04145 | Phagosome | 10 | 2.564102564 | 0.020109884 | HLA-DMA, HLA-DMB, TUBB, HLA-B, TAP2, TAP1, HLA-A, ATP6V1C1, HLA-DPA1, HLA-E |
|  |  | hsa04620 | Toll-like receptor signaling pathway | 8 | 2.051282051 | 0.024327384 | TLR1, STAT1, CTSK, IRF7, MAPK14, MAP2K7, PIK3CG, MAP2K6 |
|  |  | hsa04668 | TNF signaling pathway | 8 | 2.051282051 | 0.025456915 | CASP7, FAS, CFLAR, MAPK14, MAP2K7, MAP3K14, PIK3CG, MAP2K6 |
|  |  | hsa04310 | Wnt signaling pathway | 9 | 2.307692308 | 0.033067309 | TCF7L2, APC, FZD5, PRKCB, MYC, CUL1, DVL3, PRKCA, RBX1 |
|  |  | hsa04550 | Signaling pathways regulating pluripotency of stem cells | 9 | 2.307692308 | 0.035577196 | APC, FZD5, MYC, STAT3, DVL3, JAK2, MAPK14, FGFR2, PIK3CG |
|  |  | hsa04672 | Intestinal immune network for IgA production | 5 | 1.282051282 | 0.037409539 | IL15RA, HLA-DMA, HLA-DMB, MAP3K14, HLA-DPA1 |
|  |  | hsa04917 | Prolactin signaling pathway | 6 | 1.538461538 | 0.042464906 | STAT1, STAT3, JAK2, MAPK14, ESR2, PIK3CG |
|  |  | hsa05140 | Leishmaniasis | 6 | 1.538461538 | 0.042464906 | HLA-DMA, HLA-DMB, STAT1, JAK2, MAPK14, HLA-DPA1 |
|  |  | hsa04750 | Inflammatory mediator regulation of TRP channels | 7 | 1.794871795 | 0.049037058 | HRH1, PRKCB, PLA2G4A, PRKCA, MAPK14, PIK3CG, MAP2K6 |
|  |  | hsa05340 | Primary immunodeficiency | 4 | 1.025641026 | 0.063101069 | CIITA, BLNK, TAP2, TAP1 |
|  |  | hsa05221 | Acute myeloid leukemia | 5 | 1.282051282 | 0.064056968 | TCF7L2, MYC, STAT3, PML, PIK3CG |
|  |  | hsa04120 | Ubiquitin mediated proteolysis | 8 | 2.051282051 | 0.077371576 | UBE2H, UBE2I, UBE2N, CUL1, UBE4A, UBE2L6, PML, RBX1 |
|  |  | hsa04370 | VEGF signaling pathway | 5 | 1.282051282 | 0.082212316 | PRKCB, PLA2G4A, PRKCA, MAPK14, PIK3CG |
|  |  | hsa04064 | NF-kappa B signaling pathway | 6 | 1.538461538 | 0.085955773 | UBE2I, DDX58, BLNK, BCL10, CFLAR, MAP3K14 |
|  |  | hsa04210 | Apoptosis | 5 | 1.282051282 | 0.086118463 | CASP7, FAS, CFLAR, MAP3K14, PIK3CG |
|  |  | hsa04960 | Aldosterone-regulated sodium reabsorption | 4 | 1.025641026 | 0.087525433 | PRKCB, PRKCA, PIK3CG, NR3C2 |
|  |  | hsa04919 | Thyroid hormone signaling pathway | 7 | 1.794871795 | 0.090809379 | TBC1D4, STAT1, PRKCB, MYC, PRKCA, FOXO1, PIK3CG |
|  |  | hsa05321 | Inflammatory bowel disease (IBD) | 5 | 1.282051282 | 0.09419656 | HLA-DMA, HLA-DMB, STAT1, STAT3, HLA-DPA1 |
|  |  | hsa05230 | Central carbon metabolism in cancer | 5 | 1.282051282 | 0.09419656 | MYC, MET, FGFR2, PFKP, PIK3CG |
|  |  | hsa04630 | Jak-STAT signaling pathway | 8 | 2.051282051 | 0.09747853 | IL15RA, CTF1, STAT1, MYC, STAT2, STAT3, JAK2, PIK3CG |
|  |  | hsa04912 | GnRH signaling pathway | 6 | 1.538461538 | 0.099490266 | PRKCB, PLA2G4A, PRKCA, MAPK14, MAP2K7, MAP2K6 |

**Table 5. Functional analysis of protein–protein interaction network**

| Cell types | Diseases | Cluster | Function description | Log10(P) |
| --- | --- | --- | --- | --- |
| CD19^+^ B cells | RA | GO:0006412 | translation | -7.9 |
|  |  | GO:0043043 | peptide biosynthetic process | -7.8 |
|  |  | GO:0051345 | positive regulation of hydrolase activity | -6.8 |
|  | SLE | R-HSA-913531 | Interferon Signaling | -22.4 |
|  |  | R-HSA-909733 | Interferon alpha/beta signaling | -18.3 |
|  |  | GO:0071357 | cellular response to type I interferon | -16.4 |
| CD4^+^ T cells | RA | GO:0006397 | mRNA processing | -8.6 |
|  |  | GO:0002009 | morphogenesis of an epithelium | -6.0 |
|  |  | GO:0000398 | mRNA splicing, via spliceosome | -5.9 |
|  | SLE | R-HSA-1640170 | Cell Cycle | -16.7 |
|  |  | R-HSA-69278 | Cell Cycle, Mitotic | -16.3 |
|  |  | R-HSA-68886 | M Phase | -11.9 |
| CD14^+^ monocytes | RA | GO:0036337 | Fas signaling pathway | -6.2 |
|  |  | ko05210 | Colorectal cancer | -5.3 |
|  |  | hsa04072 | Phospholipase D signaling pathway | -5.2 |
|  | SLE | R-HSA-913531 | Interferon Signaling | -41.3 |
|  |  | R-HSA-909733 | Interferon alpha/beta signaling | -25.2 |
|  |  | GO:0034340 | response to type I interferon | -23.0 |

**Table 6.** **Module analysis in the PPI network**

| Cell types | Diseases | Mcode | Description of functions | Log10(P) | Nodes | Genes |
| --- | --- | --- | --- | --- | --- | --- |
| CD19^+^ B cells | RA | MCODE_1 | regulation of translation | -5.6 | 7 | ETF1, EIF1, IRS2, EIF1B, RASA1, ERBB2, ABCD3 |
|  |  | MCODE_1 | regulation of cellular amide metabolic process | -5.4 |  |  |
|  |  | MCODE_1 | Axon guidance | -5.3 |  |  |
|  | SLE | MCODE_1 | ISG15-protein conjugation | -9.5 | 8 | UBE2L6, HERC5, SMC4, ESR2, ISG15, ATP6V1A, IDH3A, STAT1 |
|  |  | MCODE_1 | ISG15 antiviral mechanism | -8.6 |  |  |
|  |  | MCODE_1 | Antiviral mechanism by IFN-stimulated genes | -8.4 |  |  |
|  |  | MCODE_2 | G alpha (i) signalling events | -5.5 | 3 | CXCR3, CCR2, GABBR1 |
|  |  | MCODE_2 | GPCR ligand binding | -5.3 |  |  |
| CD4^+^ T cells | RA | MCODE_1 | Estrogen signaling pathway | -6.8 | 4 | JUN, SRPK2, KRAS, HSPA1L |
|  |  | MCODE_1 | Estrogen signaling pathway | -6.3 |  |  |
|  |  | MCODE_1 | regulation of neuron apoptotic process | -5.8 |  |  |
|  | SLE | MCODE_1 | Cytosolic tRNA aminoacylation | -6.9 | 12 | DARS1, PDHA1, ALDH1A3, PRDX3, PLS3, SLC25A4, AARS1, RRM2, EEF2, NME1, MTHFD1, IARS1 |
|  |  | MCODE_1 | tRNA Aminoacylation | -6.2 |  |  |
|  |  | MCODE_1 | tRNA aminoacylation for protein translation | -6.1 |  |  |
|  |  | MCODE_2 | G2/M DNA damage checkpoint | -5.9 | 7 | AURKA, ACACA, H2BC5, HSPD1, YWHAE, CLPX, BRCA1 |
|  |  | MCODE_2 | G2/M Checkpoints | -5.1 |  |  |
|  |  | MCODE_2 | Cell Cycle | -4.9 |  |  |
|  |  | MCODE_3 | Resolution of Sister Chromatid Cohesion | -13.3 | 7 | CENPN, CCNB1, CKS2, BUB1, SGO2, CENPE, SKA1 |
|  |  | MCODE_3 | Mitotic Prometaphase | -12 |  |  |
|  |  | MCODE_3 | Mitotic Anaphase | -11.6 |  |  |
| CD14^+^ monocytes | RA | MCODE_1 | Axon guidance | -3.2 | 9 | KRAS, HNRNPR, SRPRB, TUBA1A, PRPF6, RPA2, PSEN1, EWSR1, SSR1 |
|  |  | MCODE_1 | Nervous system development | -3.2 |  |  |
|  | SLE | MCODE_1 | Antigen Presentation: Folding, assembly and peptide loading of class I MHC | -15.6 | 12 | TAP2, HLA-B, TAP1, PNN, SRRM2, HLA-A, SEC24A, HLA-E, TMEM41B, RBM8A, SRPK2, IFI16 |
|  |  | MCODE_1 | antigen processing and presentation of endogenous peptide antigen | -13.5 |  |  |
|  |  | MCODE_1 | antigen processing and presentation of endogenous peptide antigen via MHC class I | -13.5 |  |  |
|  |  | MCODE_2 | Cyclin E associated events during G1/S transition | -5.7 | 9 | TUBB, CUL1, GART, PSMD14, ACSL3, DLAT, FBL, MYC, EEF2 |
|  |  | MCODE_2 | Cyclin A:Cdk2-associated events at S phase entry | -5.6 |  |  |
|  |  | MCODE_2 | G1/S Transition | -5.1 |  |  |
|  |  | MCODE_3 | regulation of growth | -4.9 | 7 | NUP205, STAT3, SGPL1, IFITM3, ESR2, PML, TOMM20 |
|  |  | MCODE_3 | Interferon Signaling | -4.9 |  |  |
|  |  | MCODE_3 | protein import | -4.9 |  |  |
|  |  | MCODE_4 | Asparagine N-linked glycosylation | -6.7 | 6 | ARF4, VCP, CYLD, PFKP, RPS27A, COPB2 |
|  |  | MCODE_4 | ER to Golgi vesicle-mediated transport | -5.1 |  |  |
|  |  | MCODE_4 | protein deubiquitination | -4.7 |  |  |
|  |  | MCODE_5 | negative regulation of multi-organism process | -7.6 | 6 | PPM1B, ZC3HAV1, ARRB1, ISG15, PPARG, ANXA2 |
|  |  | MCODE_5 | regulation of multi-organism process | -6.1 |  |  |
|  |  | MCODE_5 | positive regulation of endocytosis | -5.5 |  |  |
|  |  | MCODE_6 | ISG15 antiviral mechanism | -10.4 | 4 | UBE2L6, STAT1, HERC5, DDX58 |
|  |  | MCODE_6 | Antiviral mechanism by IFN-stimulated genes | -10.2 |  |  |
|  |  | MCODE_6 | regulation of type I interferon production | -9.4 |  |  |
|  |  | MCODE_7 | Peptide ligand-binding receptors | -8.6 | 4 | CCL16, CCR1, FPR2, NPY2R |
|  |  | MCODE_7 | Class A/1 (Rhodopsin-like receptors) | -7.7 |  |  |

**Table 7. Currently available MAPK and PI3kinase inhibitors**

| Kinase | Inhibitor | Chemical formula | Target | Indication | Status | References |
| --- | --- | --- | --- | --- | --- | --- |
| MAPK | VX-702 | C_19_H_12_F_4_N_4_O_2_ | p38α | RA, Acute coronary syndrome | Phase II (2005) | [1^,^ 2] |
|  | Doramapimod（BIRB -796） | C_31_H_37_N_5_O_3_ | p38α | Psoriasis, RA, Crohn’s | Phase II (2003) | [3^,^ 4] |
|  | Talmapimod (SCIO-469) | C₂₇H₃₀ClFN₄O₃ | p38α | RA, IBD, Multiple myeloma, Dental pain | Phase II (2005) | [4^,^ 5] |
|  | RWJ-67657 | C₂₇H₂₄FN₃O | p38α, p38β | RA | Phase I (discontinued) | [6] |
|  | TAK-715 | C₂₄H₂₁N₃OS | p38α, p38β | RA | Phase II (2005) | [7] |
|  | Dilmapimod (SB-681323) | C₂₃H₁₉F₃N₄O₃ | TNF-α | RA, COPD, Atherosclerosis, Neuropathic pain | Phase II (2006) | [8^,^ 9] |
|  | FR167653 | C₂₄H₂₀FN₅O₆S | TNF-α, IL-1β | RA | Pre-clinical study | [10] |
|  | VX-745 | C₁₉H₉Cl₂F₂N₃OS | p38α | RA, Alzheimer's disease | Discontinued | [11^,^ 12] |
|  | Pamapimod (R-1503) | C_19_H_20_F_2_N_4_O_4_ | p38α, p38β | RA | Phase II (2006) | [13] |
|  | BMS-582949 (PS540446) | C_22_H_26_N_6_O_2_ | p38α | RA, Psoriasis, Atherosclerosis | Phase II (2007) | [14] |
|  | AMG-548 | C₂₉H₂₇N₅O | p38α | RA, COPD | Phase I (discontinued) | [15] |
|  | PH-797804 | C_22_H_19_BrF_2_N_2_O_3_ | p38α | RA, Osteoarthritis, Neuralgia, COPD | Phase II (2007) | [16] |
| PI3K | IC-87114 | C_22_H_19_N_7_O | PI3Kδ | SLE, COPD, RA | Pre-clinical study | [17-19] |
|  | AS-605240 | C_12_H_7_N_3_O_2_S | PI3Kγ | SLE, RA, COPD, Atherosclerosis | Pre-clinical study | [20^,^ 21] |
|  | CZC24832 | C_15_H_17_FN_6_O_2_S | PI3Kγ | SLE, RA | Pre-clinical study | [19^,^ 22] |
|  | Parsaclisib (INCB050465) | C_20_H_22_ClFN_6_O_2_ | PI3Kδ | SLE, Sjögren’s syndrome, Autoimmune hemolytic anaemia | Pre-clinical study | [23] |

RA: Rheumatoid arthritis; IBD: Inflammatory bowel disease; COPD: Chronic obstructive pulmonary disease; SLE: Systemic lupus erythematosus.





Supplementary Figure 1. GO analysis of CD19^+^ B cells in RA and SLE. (A) Highly significant GO terms identified in the biological processes, cellular components and molecular functions of RA and (B) SLE. GO, Gene Ontology.





Supplementary Figure 2. GO analysis of CD4^+^ T cells in RA and SLE. (A) Highly significant GO terms identified in the biological processes, cellular components and molecular functions of RA and (B) SLE. GO, Gene Ontology.

***

***

Supplementary Figure 3. GO analysis of CD14^+^ monocytes in RA and SLE. (A) Highly significant GO terms identified in the biological processes, cellular components and molecular functions of RA and (B) SLE. GO, Gene Ontology.





Supplementary Figure 4. The KEGG pathway analysis of CD19^+^ B-, CD4^+^ T- cells and CD14^+^ monocytes in RA and SLE. (A) MAPK and (B) PI3K signaling pathways were enriched in CD19^+^ B cells of RA and SLE, respectively. (B) MAPK and (C) Focal adhesion signaling pathways were enriched in CD4^+^ T cells of RA and SLE, respectively (C) PI3K signaling pathway was enriched in CD14^+^ monocytes of both RA and SLE. Up-regulated, down-regulated and unchanged genes were denoted in red, green and grey color.

**References**

1. Kuliopulos A, Mohanlal R, Covic L. Effect of selective inhibition of the p38 MAP kinase pathway on platelet aggregation. *Thromb Haemost* (2004) **92**: 1387-1393.

2. Ding C. Drug evaluation: VX-702, a MAP kinase inhibitor for rheumatoid arthritis and acute coronary syndrome. *Curr Opin Investig Drugs* (2006) **7**: 1020-1025.

3. Behr TM, Berova M, Doe CP, Ju H, Angermann CE, Boehm J *et al*. p38 mitogen-activated protein kinase inhibitors for the treatment of chronic cardiovascular disease. *Curr Opin Investig Drugs* (2003) **4**: 1059-1064.

4. Schreiber S, Feagan B, D'Haens G, Colombel JF, Geboes K, Yurcov M *et al*. Oral p38 mitogen-activated protein kinase inhibition with BIRB 796 for active Crohn's disease: a randomized, double-blind, placebo-controlled trial. *Clin Gastroenterol Hepatol* (2006) **4**: 325-334.

5. Vanderkerken K, Medicherla S, Coulton L, De Raeve H, Willems A, Lawson M *et al*. Inhibition of p38alpha mitogen-activated protein kinase prevents the development of osteolytic bone disease, reduces tumor burden, and increases survival in murine models of multiple myeloma. *CANCER RES* (2007) **67**: 4572-4577.

6. Westra J, Limburg PC, de Boer P, van Rijswijk MH. Effects of RWJ 67657, a p38 mitogen activated protein kinase (MAPK) inhibitor, on the production of inflammatory mediators by rheumatoid synovial fibroblasts. *ANN RHEUM DIS* (2004) **63**: 1453-1459.

7. Miwatashi S, Arikawa Y, Kotani E, Miyamoto M, Naruo K, Kimura H *et al*. Novel inhibitor of p38 MAP kinase as an anti-TNF-alpha drug: discovery of N-[4-[2-ethyl-4-(3-methylphenyl)-1,3-thiazol-5-yl]-2-pyridyl]benzamide (TAK-715) as a potent and orally active anti-rheumatoid arthritis agent. *J MED CHEM* (2005) **48**: 5966-5979.

8. Singh D, Smyth L, Borrill Z, Sweeney L, Tal-Singer R. A randomized, placebo-controlled study of the effects of the p38 MAPK inhibitor SB-681323 on blood biomarkers of inflammation in COPD patients. *J CLIN PHARMACOL* (2010) **50**: 94-100.

9. Goh FG, Midwood KS. Intrinsic danger: activation of Toll-like receptors in rheumatoid arthritis. *Rheumatology (Oxford)* (2012) **51**: 7-23.

10. Nishikawa M, Myoui A, Tomita T, Takahi K, Nampei A, Yoshikawa H. Prevention of the onset and progression of collagen-induced arthritis in rats by the potent p38 mitogen-activated protein kinase inhibitor FR167653. *Arthritis Rheum* (2003) **48**: 2670-2681.

11. Duffy JP, Harrington EM, Salituro FG, Cochran JE, Green J, Gao H *et al*. The Discovery of VX-745: A Novel and Selective p38alpha Kinase Inhibitor. *ACS MED CHEM LETT* (2011) **2**: 758-763.

12. Alam J, Blackburn K, Patrick D. Neflamapimod: Clinical Phase 2b-Ready Oral Small Molecule Inhibitor of p38alpha to Reverse Synaptic Dysfunction in Early Alzheimer's Disease. *J Prev Alzheimers Dis* (2017) **4**: 273-278.

13. Alten RE, Zerbini C, Jeka S, Irazoque F, Khatib F, Emery P *et al*. Efficacy and safety of pamapimod in patients with active rheumatoid arthritis receiving stable methotrexate therapy. *ANN RHEUM DIS* (2010) **69**: 364-367.

14. Norman P. BMS-582949: crystalline form of a p38alpha inhibitor? WO2008079857. *EXPERT OPIN THER PAT* (2009) **19**: 1165-1168.

15. Lee MR, Dominguez C. MAP kinase p38 inhibitors: clinical results and an intimate look at their interactions with p38alpha protein. *CURR MED CHEM* (2005) **12**: 2979-2994.

16. Selness SR, Devraj RV, Devadas B, Walker JK, Boehm TL, Durley RC *et al*. Discovery of PH-797804, a highly selective and potent inhibitor of p38 MAP kinase. *BIOORG MED CHEM LETT* (2011) **21**: 4066-4071.

17. Wang Y, Zhang L, Wei P, Zhang H, Liu C. Inhibition of PI3Kdelta improves systemic lupus in mice. *INFLAMMATION* (2014) **37**: 978-983.

18. Marahatta A, Bhandary B, Lee YC, Kim SR, Chae HJ. Development and validation of a highly sensitive LC-MS/MS method for quantification of IC87114 in mice plasma, bronchoalveolar lavage and lung samples: Application to pharmacokinetic study. *J Pharm Biomed Anal* (2014) **89**: 197-202.

19. Mina-Osorio P, LaStant J, Keirstead N, Whittard T, Ayala J, Stefanova S *et al*. Suppression of glomerulonephritis in lupus-prone NZB x NZW mice by RN486, a selective inhibitor of Bruton's tyrosine kinase. *Arthritis Rheum* (2013) **65**: 2380-2391.

20. Camps M, Ruckle T, Ji H, Ardissone V, Rintelen F, Shaw J *et al*. Blockade of PI3Kgamma suppresses joint inflammation and damage in mouse models of rheumatoid arthritis. *NAT MED* (2005) **11**: 936-943.

21. Barber DF, Bartolome A, Hernandez C, Flores JM, Redondo C, Fernandez-Arias C *et al*. PI3Kgamma inhibition blocks glomerulonephritis and extends lifespan in a mouse model of systemic lupus. *NAT MED* (2005) **11**: 933-935.

22. Hernandez-Florez D, Valor L. Protein-kinase inhibitors: A new treatment pathway for autoimmune and inflammatory diseases? *Reumatol Clin* (2016) **12**: 91-99.

23. Forero-Torres A, Ramchandren R, Yacoub A, Wertheim MS, Edenfield WJ, Caimi P *et al*. Parsaclisib, a potent and highly selective PI3Kdelta inhibitor, in patients with relapsed or refractory B-cell malignancies. *BLOOD* (2019) **133**: 1742-1752.
